# A new method for roadheader pick arrangement based on meshing pick spatial position and rock cutting verification

**DOI:** 10.1371/journal.pone.0260183

**Published:** 2021-11-17

**Authors:** Mengqi Zhang, Xianguo Yan, Guoqiang Qin

**Affiliations:** 1 School of Mechanical Engineering, Taiyuan University of Science and Technology, Taiyuan, Shanxi Province, China; 2 Shanxi Institute of Energy, Taiyuan, Shanxi Province, China; 3 National Engineering Laboratory for Coal Mining Machinery, Taiyuan, Shanxi Province, China; University of Vigo, SPAIN

## Abstract

This paper proposes a cutting head optimization method based on meshing the spatial position of the picks. According to the expanded shape of the spatial mesh composed of four adjacent picks on the plane, a standard mesh shape analysis method can be established with mesh skewness, mesh symmetry, and mesh area ratio as the indicators. The traversal algorithm is used to calculate the theoretical meshing rate, pick rotation coefficient, and the variation of cutting load for the longitudinal cutting head with 2, 3, and 4 helices. The results show that the 3-helix longitudinal cutting head has better performance. By using the traversal result with maximum theoretical meshing rate as the design parameter, the longitudinal cutting head CH51 with 51 picks was designed and analyzed. The prediction model of pick consumption is established based on cutting speed, direct rock cutting volume of each pick, pick rotation coefficient, uniaxial compressive strength, and CERCHAR abrasivity index. And the rock with normal distribution characteristics of Uniaxial Compressive Strength is used for the specific energy calculating. The artificial rock wall cutting test results show that the reduction in height loss suppresses the increase in pick equivalent loss caused by the increase in mass loss, and the pick consumption in this test is only 0.037–0.054 picks/m^3^. In addition, the correlation between the actual pick consumption and the prediction model, and the correlation between the actual cutting specific energy and the theoretical calculation value are also analyzed. The research results show that the pick arrangement design method based on meshing pick tip spatial position can effectively reduce pick consumption and improve the rock cutting performance.

## 1. Introduction

**Fig 1:**
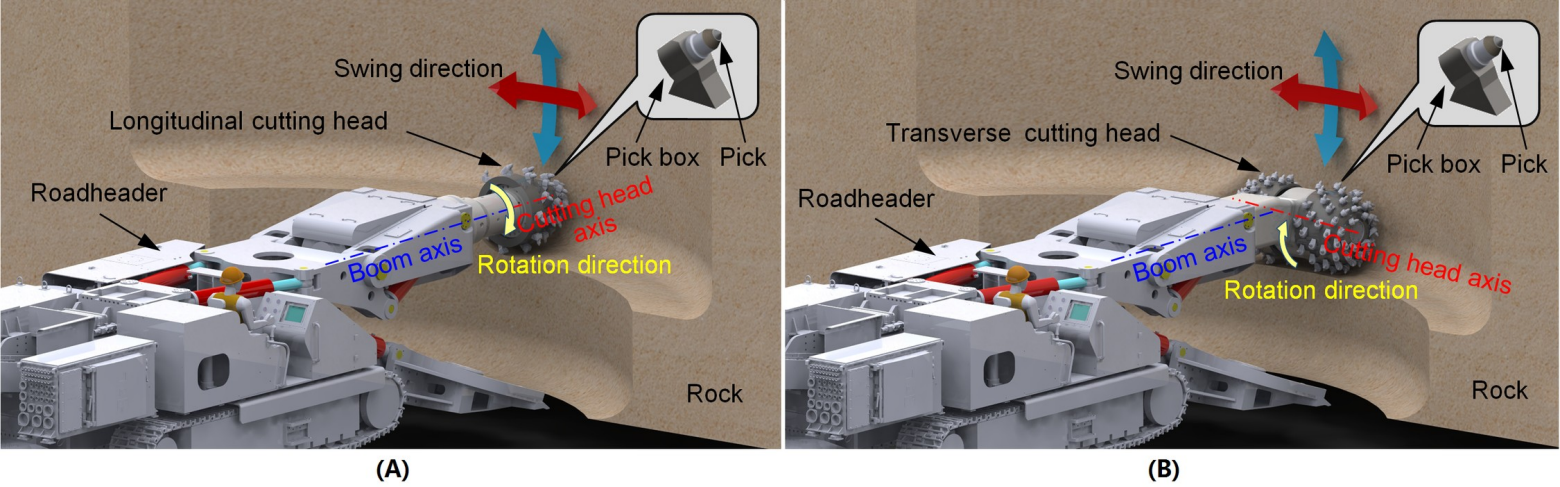
Two types of cutting head. (A) Milling process with longitudinal cutting head. (B) Ripping process with transverse cutting Head.

Boom-type roadheader is one of the critical mining equipment. It can be divided into the longitudinal and transverse types according to relative installation form of the cutting head and the cutter boom. For the roadheader with a longitudinal cutting head, the rotation axis of the cutting head coincides with the center of the cutting boom, and the pick cuts the rock with milling process, as shown in Fig 1A. The picks are usually arranged with one pick on one cutting line. On the same cutting line, the pick can effectively cut the fresh rock surface. However, due to the reaction force of the cutting head is perpendicular to the gravity direction of the roadheader, the lateral slip tends to occur during the swing process. Therefore, the longitudinal cutting head is mainly used for rapid excavation of conventional roadways with uniaxial compressive strength (UCS) is not more than 100MPa [1]. The cutting heads for the transverse roadheader are arranged symmetrically on both sides of the cutting boom, the rotation axis is perpendicular to the boom, and the picks cut the rock in a ripping way, as shown in Fig 1B. The picks are usually arranged with multi-picks on one cutting line, and each pick on the same cutting line can repeat to increase the depth of the same cutting groove. When the adjacent cutting groove reaches a certain depth, the micro-cracks at the bottom of the cutting chips are connected, causing the cutting clips to fall from the hard rock mass. In addition, due to the roadheader weight being consistent with the cutting reaction force, the roadheader weight also promotes the deepening of the cutting groove during the cutting process. Therefore, the roadheader with a transverse cutting head is mainly used for higher hardness rock excavation [2, 3],as shown in Fig 1. Picks are installed in the pick boxes and cutting into the rock in sequence during the cutting head rotation, and the rock is broken by the combination of compression and shear [4]. The picks are arranged on the surface of the cutting head in the form of a unique helix. To be suitable for drilling and swing, the cutting head of the roadheader is usually designed as a combination of the cylinder, cone, and sphere, or fitted by these basic geometries, which does not have the condition that the cylindrical cutting drum of shearer can develop into a plane along the generatrix [5]. Moreover, the mechanical properties of the rock for the roadheader are generally higher than coal cutting for shearer, and the cutting chips are also different. Therefore, the traditional pick arrangement method for the drum is not suitable for the design and analysis of the cutting head with a complex spatial structure [6–8]. The pick spatial position and attitude are the main factors affecting pick consumption and rock cutting performance for the cutting head [9–13]. When the characteristic size of the cutting head is confirmed, the pick spatial position can be determined by the two parameters of the helix shape and the cutting line spacing [14–16]. In the conventional design process, the helix geometry is determined first, and then the picks are arranged on the helix with appropriate spacing according to rock properties. However, this design method often leads to excessive concentration of picks in a certain circumferential angle or non-uniform pick arrangement in the local area where the helix spacing changes significantly [17]. For the picks on the cutting head with an appropriate attack angle, when the cutting line spacing does not match the circumferential angular interval, the pick arrangement will be non-uniform. In some cases of rock cutting, pick consumption more than 0.2 picks per cubic meter; for some rocks with higher uniaxial compressive strength and CERCHAR abrasivity index, it can even reach 1.0 picks per cubic meter, which seriously affects the working performance of the roadheader [18, 19]. Many studies have pointed out that a reasonable ratio of the cutting line spacing to the cutting depth and an appropriate attack angle can significantly reduced the cutting force for the wear of picks [20, 21]. However, for the cutting head with continuous changes of cross-section and multiple working picks in the rock cutting zone, the research on pick arrangement uniformity and cutting performance caused by helix geometry and cutting line spacing are insufficient.

For getting the uniform pick arrangement and improving rock cutting performance, this paper established a new design method for the cutting head based on meshing pick spatial position, and verified the advantage of working performance for the optimized cutting head in pick consumption and specific energy by rock cutting test. As an example with the longitudinal cutting head, the pick arrangement meshing rate was proposed to evaluate the relative position between picks and helices. The optimized pick arrangement results were obtained with the maximum number of standard mesh cells from all pick arrangement schemes by the traversal algorithm. The working performance of the cutting head by using the optimized pick arrangement is higher than other cutting heads, which verified the feasibility of the new design method. Based on the prediction model of pick consumption and the calculation model of specific energy, the working performance of the cutting head is analyzed. The milling processes of the rock cutting test shows that the pick consumption of the cutting head designed by meshing the spatial position of the picks is lower. The rock cutting test data of pick consumption and specific energy are correlated with the prediction results. The reasonableness of this cutting head design method was verified by rock cutting performance.

## 2. Meshing method of pick position for the cutting head

### 2.1 Meshing method and morphology quality index

**Fig 2:**
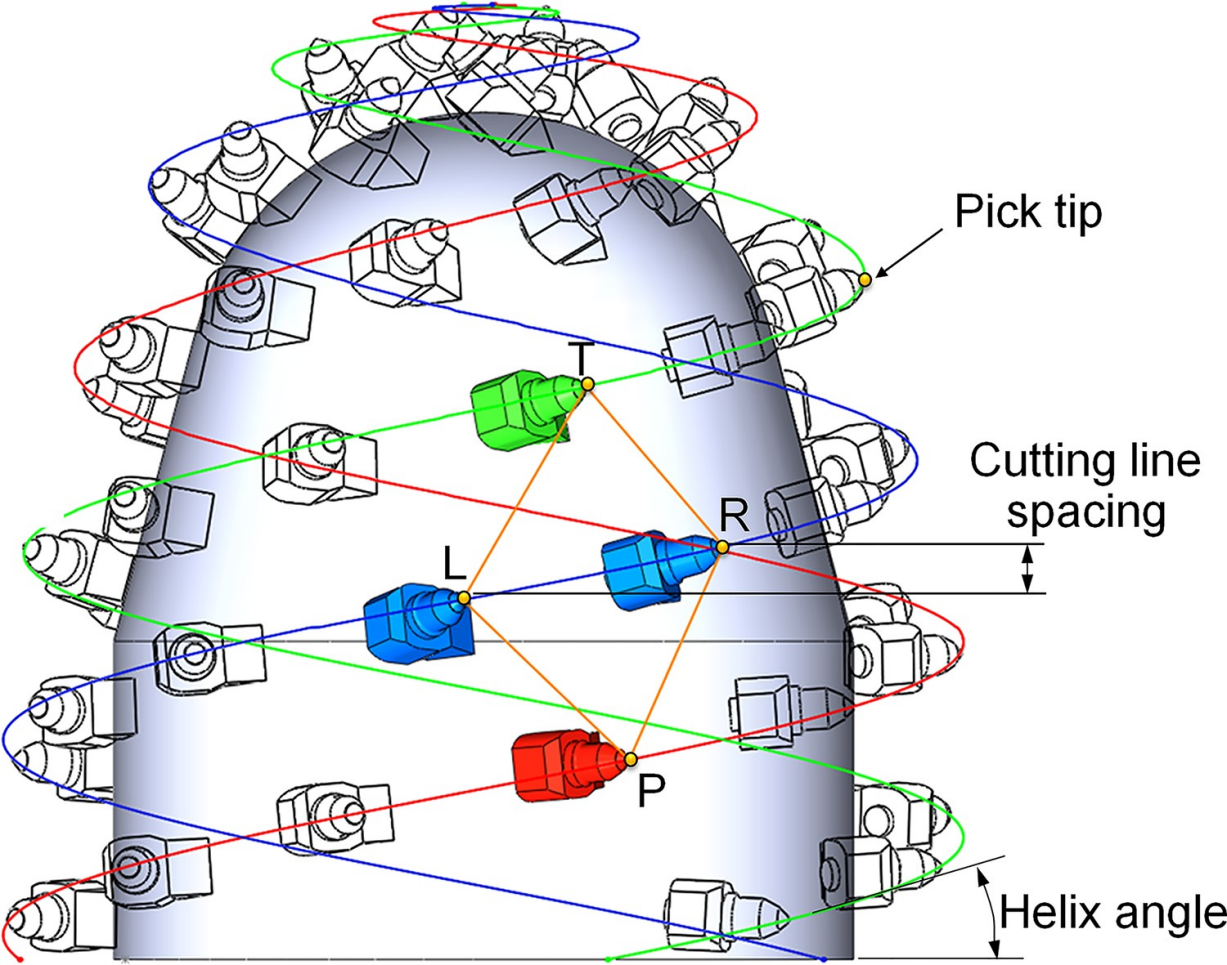
Mesh cell structure of Cutting head.


Cutting head surface can be divided into multiple non-intersecting spatial quadrilateral mesh cells with the tips of the adjacent picks as the vertex to make picks have uniform axial position and circumferential position, as shown in Fig 2. In anyone of the mesh cell P-L-T-R with vertex P as the marked point, the cutting line spacing is used to determine the positions of adjacent vertex L and R along the cutting head axis on the same helix. In combination with the helix angle that controls helix geometry, the circumference position of vertex L and R can be determined. Vertex T is located on the other side of the helix where vertices L and R are located, and it is another top vertex of the mesh cell. The helix parameter of a cylinder, cone, and sphere are shown in Eqs (1), (2) and (3), respectively [22].

$$\left\{ \begin{array}{l} x_{\mathrm{cy}}=r\cos\theta \\ y_{\mathrm{cy}}=r\sin\theta \\ z_{\mathrm{cy}}=r\theta \tan\varepsilon \end{array}\right.$$ (1)

$$\left\{ \begin{array}{l} x_{co}=\rho \cos\theta e^{\theta \sin\psi \tan\varepsilon }\\ y_{co}=\rho \sin\theta e^{\theta \sin\psi \tan\varepsilon }\\ z_{co}=\rho \cot\psi e^{\theta \sin\psi \tan\varepsilon } \end{array}\right.$$ (2)

$$\left\{ \begin{array}{l} x_{sp}=\frac{2r}{1+A^{2}e^{-2\theta \tan\varepsilon }}\cos\theta Ae^{-\theta \tan\varepsilon }\\ y_{sp}=\frac{2r}{1+A^{2}e^{-2\theta \tan\varepsilon }}\sin\theta Ae^{-\theta \tan\varepsilon }\\ z_{sp}=\frac{r}{1+A^{2}e^{-2\theta \tan\varepsilon }}\times (1-A^{2}e^{-2\theta \tan\varepsilon }) \end{array}\right.$$ (3)

Where *x*_*cy*_, *y*_*cy*_, *z*_*cy*_ are point coordinates of cylinder helix; *x*_*co*_, *y*_*co*_, *z*_*co*_ are point coordinates of cone helix; and *x*_*sp*_, *y*_*sp*_, *z*_*sp*_ are point coordinates of sphere helix. *r* is the radius of a point on the helix, *mm*. *θ* is the circumferential angle of a point on the helix, rad. *ε* is the helix angle, rad. *ρ* is the polar diameter of the cone, mm. *ψ* is half-angle of the cone, rad. *A* is a constant which is equal to 1 for the sphere.

The geometry of the mesh cell is determined by helix parameters and cutting line spacing. When the position of any pick of a mesh cell changes on the helix, the shape of the mesh cell with the pick tip as the vertex will change accordingly, causing the mesh cell shape in the local area of the cutting head to change. Therefore, according to the geometric shape of each quadrilateral mesh cell, the reasonable matching of the helix parameters and the design parameters of cutting line spacing can be analyzed to obtain a uniform pick arrangement. This method can avoid changes in the shape and geometric dimensions of the cutting head on the uniformity of pick arrangement.

Along the L-R line of pick tip on the same helix, the spatial quadrilateral mesh cell can be divided into upper triangular piece△LRT and lower triangular piece△LRP. A new quadrilateral PRTL can be formed by rotating △LRT along the L-R line to the plane where △LRP is located. Therefore, the pick arrangement mesh can be defined as a flat quadrilateral composed of adjacent picks on the same helix and the adjacent helix.

**Fig 3:**
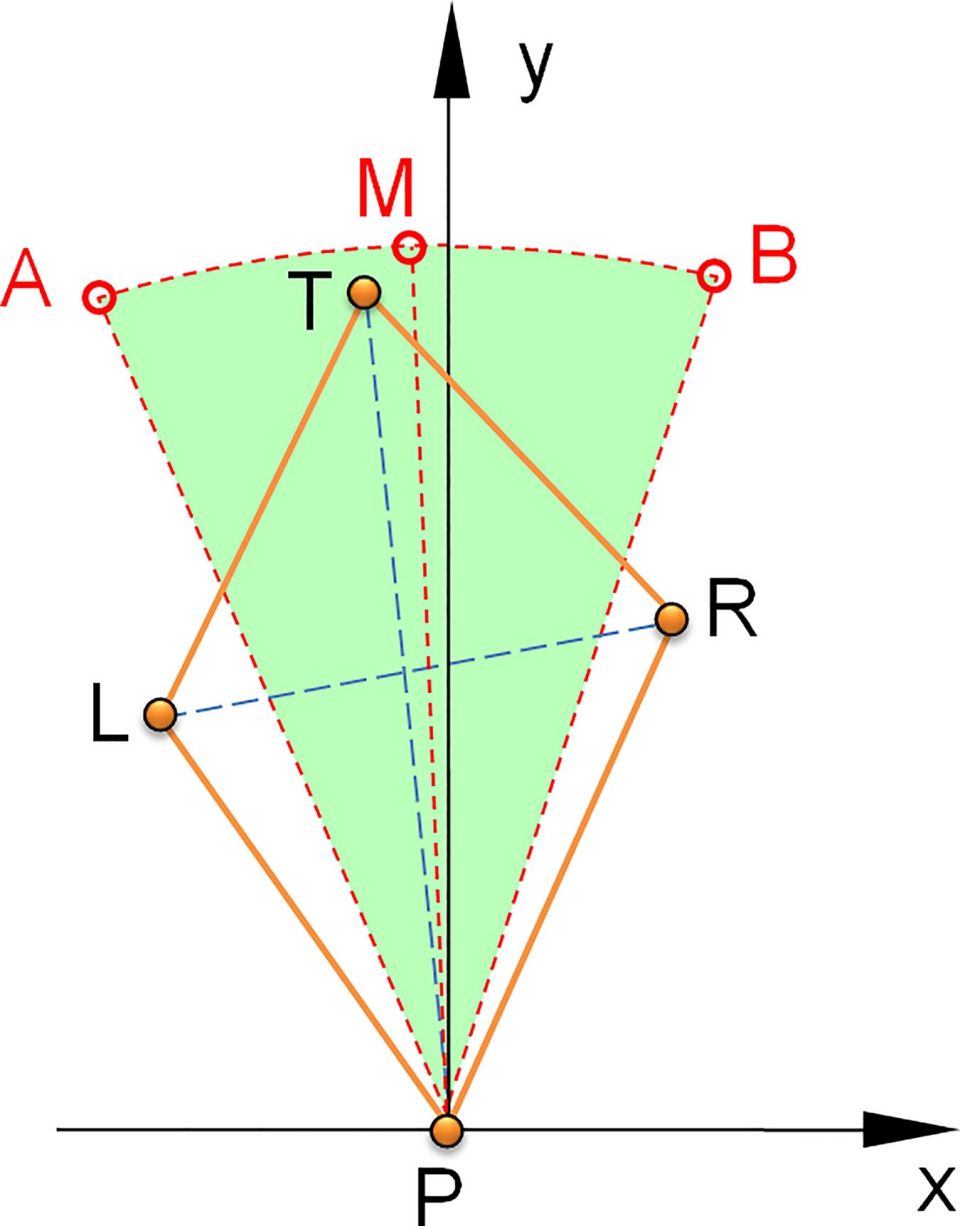
Calculation principle for the pick arrangement meshing method.

According to the geometric characteristics of the mesh cell on the plane, mesh area ratio, mesh symmetry degree, and mesh skewness are proposed to evaluate the performance of the pick arrangement mesh in this paper, as shown in Fig 3.

The mesh skewness indicates the deviation of the longitudinal-diagonal P-T of a mesh cell from the average value of the helix angle of the cutting head. It is represented by S_k_ and is used to determine the uniformity of the pick positions on the spaced helices. In x-y coordinate, PM is the average value of the helix angle, ∠MPA and ∠MPB are the allowable skew angles that range along forward and reverse the longitudinal-diagonal direction, respectively. The range of S_k_is from ∠xPM-∠MPB to ∠xPM+∠MPA, and can be expressed by Eq (4).


$$∠xPB\leq S_{k}\leq ∠xPA$$ (4)

The mesh symmetry is the ratio of two sides length of the upper and lower triangle elements used to analyze the uniformity of the pick arrangement on the adjacent helix. For the lower triangular piece, mesh symmetry is the ratio of the small value to the large value of P_L_ and P_R_, and can be denoted as S_yP_, see Eq (5). For the upper triangular piece, mesh symmetry can be denoted as S_yT_ by the ratio of the small value to the large value of T_L_ and T_R_, see Eq (6). In the same mesh cell, when S_yT_ and S_yP_ are both close to 1, vertex P and T are located near the mid-vertical line of vertex L and R on the adjacent helix lines, and the adjacent picks can be arranged uniformly on the circumference. The pick count interacting with the rock keeps small changes, which can avoid significant cutting load fluctuation.


$$S_{yP}=\min(PL,PR)/\max(PL,PR)$$ (5)

$$S_{yT}=\min(TL,TR)/\max(TL,TR)$$ (6)

The Mesh area ratio is the proportion of the triangular piece area that makes up the quadrilateral mesh cell. Mesh area ratio of upper triangular △LRT to lower triangular △LRP, denoted by A_TP_, is used to analyze the matching of helix and cutting line spacing, see Eq (7). In addition, to avoid the deformed mesh cell, the area ratio A_LR_ of left piece △TPL to right piece △TPR is used as another indicator of the mesh area ratio, see Eq (8). When picks are arranged uniformly, A_TP_ and A_LR_ are approximately equal, and the values are both close to 1.


$$A_{TP}=S_{△LRT}/S_{△LRP}$$ (7)

$$A_{LR}=S_{△TPL}/S_{△TPR}$$ (8)

### 2.2 Pick arrangement meshing rate

**Fig 4:**
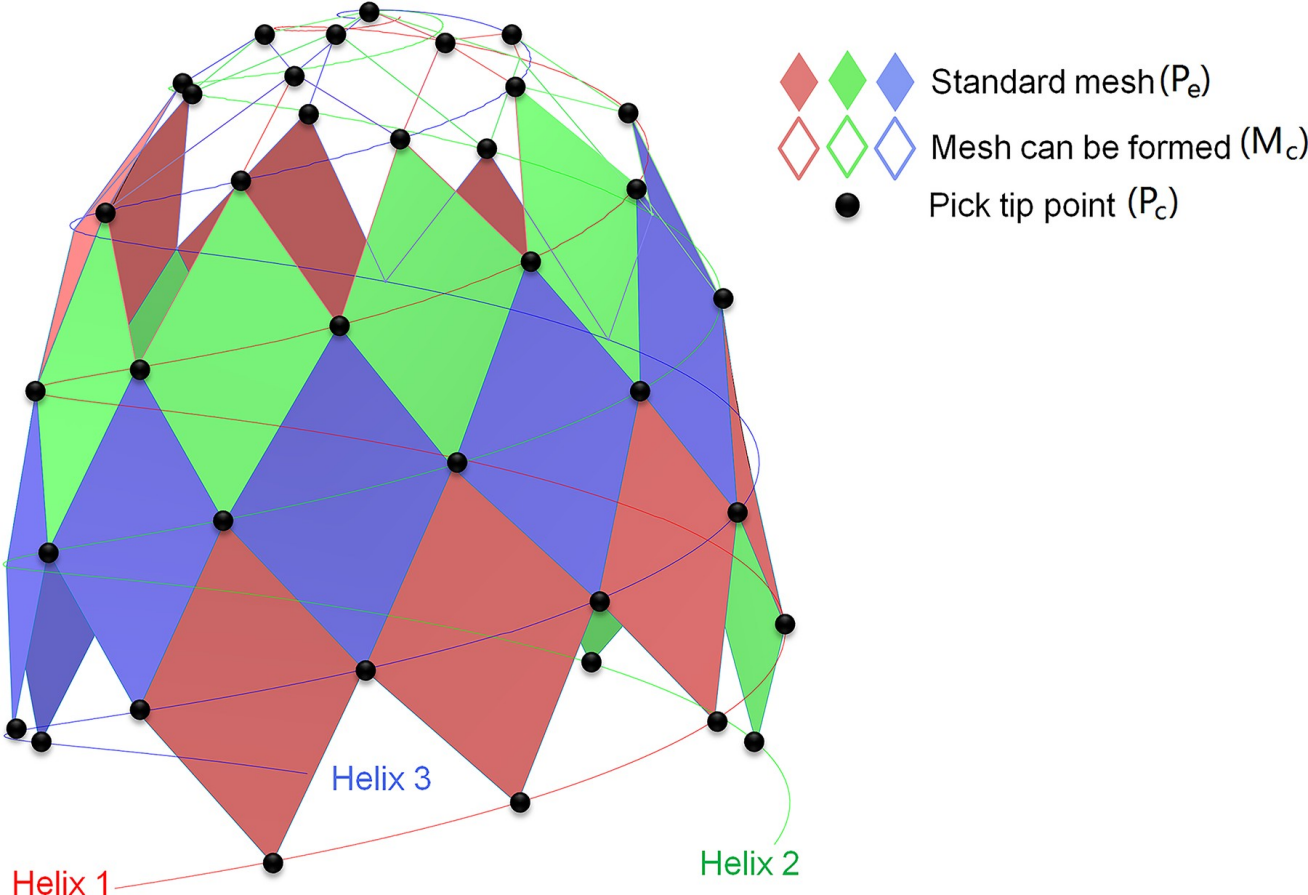
Meshes on the cutting head.

Among all non-intersecting meshes formed by adjacent picks, the pick arrangement mesh meeting the above indicators can be defined as standard mesh, as shown in Fig 4. The more standard mesh cells, the more uniform the pick arrangement.

RMR is defined as the percentage of standard mesh cells to the mesh cells count, AMR is defined as the percentage of standard mesh cells to the picks count. And they can be calculated by Eq (9) and Eq (10), respectively.


$$RMR=\frac{P_{e}}{M_{c}}$$ (9)

$$AMR=\frac{P_{e}}{P_{c}}$$ (10)

Where *P*_*e*_ is the standard mesh count. *M*_*c*_ is the mesh count that can be formed. *P*_*c*_ is the picks count on the cutting head.

However, when RMR is used as the index of the uniformity for pick arrangement, a larger RMR may be obtained with less count of picks and uniformed pick arrangement. In this case, the pick count is less than the conventional design result, and the distance between each pick along the helix and cutting head axis is more significant. The adjacent picks cannot work in unrelieved cutting mode, and the cutting force and normal force on picks are higher than that of relieved cutting mode, which results in the pick wear increases. Thus, the picks for rock cutting need to change frequently, which is not the optimal design result. When AMR is used as the uniformity index for pick arrangement, the relationship between standard mesh cells and pick arrangement mesh count cannot be accurately expressed. For these reasons, according to the effect of picks count and helix numbers on the mesh cell formed, this paper proposes to use TMR as the indicator of pick arrangement uniformity. TMR is the percentage of the standard mesh to the theoretical maximum mesh count formed by all picks and can be used to assess the pick arrangement uniformity on the surface of the cutting head and the variation of cutting load, and can be calculated by Eq (11),

$$TMR=\frac{P_{e}}{P_{c}-h-1}$$ (11)

Where *h* is helices count.

### 2.3 Traversal algorithm of pick arrangement for the cutting head

**Fig 5:**
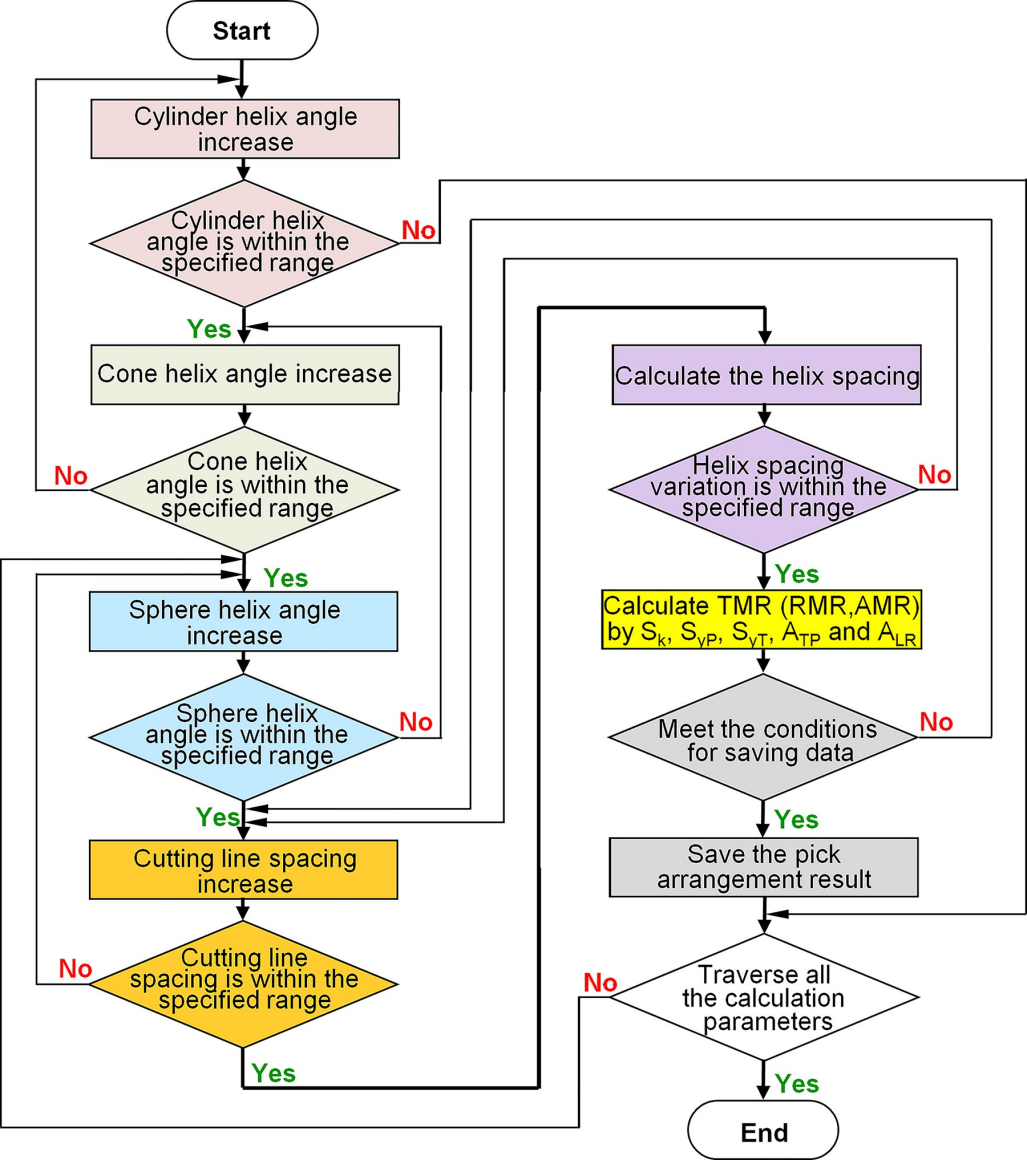
Traversal algorithm block diagram of pick arrangement.

The pick spatial position is determined by helix geometry and cutting line spacing for the cutting heads with defined shape and geometric characteristics. The traversal algorithm calculates all parameters combinations of the helix geometry and cutting line spacing to obtain a uniform pick arrangement. In the nested loop structure for pick arrangement, from outer layer to inner layer, the helix angles of the cylinder, cone, and sphere are taken as independent circulation variables in turn; the variation degree of the distance between adjacent helix is taken as the constraint condition. The TMR of all pick arrangement results is calculated within the reasonable value range of the cutting line spacing, and the pick arrangement parameters and TMR values that meet the constraint conditions are saved. The traversal algorithm block diagram is shown in Fig 5. As the helix angle of the inner loop exceeds the set range or the adjacent helix distance exceeds the set threshold, the loop jumps to the outer layer helix angle calculation. The traversal algorithm automatically increases the initial helix angle of the outer layer geometry; then, the traversal calculation of the helix geometry and the cutting line spacing increase for the inner layer geometry will be performed again.

**Fig 6:**
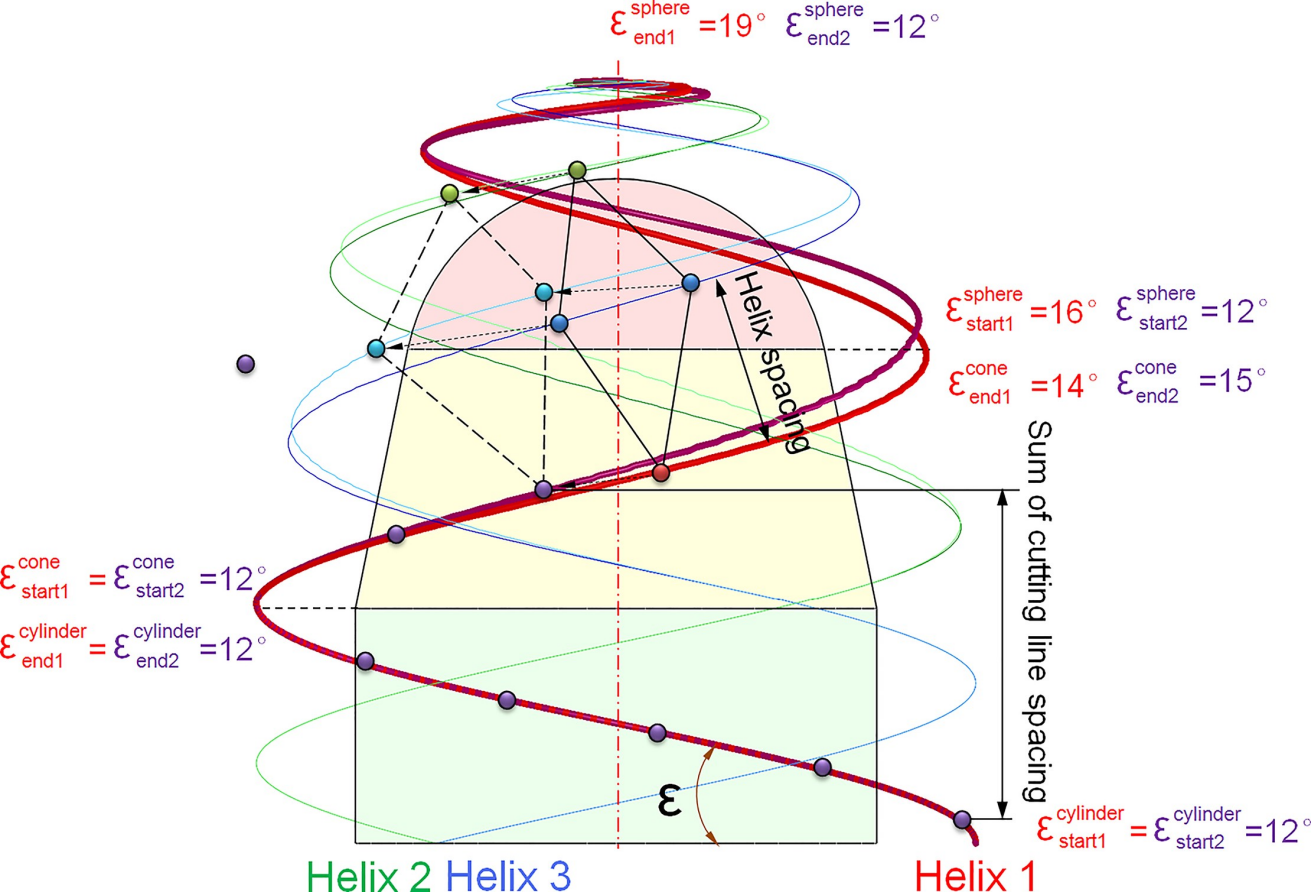
Traversal algorithm block diagram of pick arrangement.

For instance, the calculation range of the helix angle for the cylinder, cone, and sphere is usually 12–20°, the range of cutting line spacing is 20-35mm. Both the helix start and end angle of the cylinder are 12°, the current helix start and end angle for the outer layer cone are 12° and 14°, respectively; The helix start and end angle for the inner layer sphere are 16° and 19°, respectively. Within the process, each pick position changes in the specified range of cutting line spacing. If the variation of helix spacing is beyond the permitted threshold of the constraint condition, the traversal calculation will jump from the sphere to the cone, as shown in Fig 6. For example, if the continuous change value of the helix spacing in the transition area from the cone to the sphere exceeds3%, the loop will jump to the next helix angle end value. According to the loop step value, the helix end angle for the cone is automatically increased by 1° to 15°. At the same time, the helix start and end angle for the sphere will be re-initialized to 12°. If the variation of helix spacing is within the permitted threshold, the TMR will be recalculated; otherwise, the helix start and end angle of the inner sphere will continue to increase, and the above loop process will be repeated. Suppose the helix end angel of the cone beyond 20° after jumped, in that case, the helix start angle for the cone will increase 1° to 13° automatically, and the sphere start and end angle are reset to 12° for the subsequent traversal calculation.

## 3. The longitudinal cutting head design

### 3.1 The influence of helix on pick arrangement

To verify the effectiveness of the pick position meshing method for cutting head design, the longitudinal-axis cutting head of the EBZ260W roadheader was designed in detail. The roadheader is a medium-to-heavy mining machine with a rotation speed of 32.5rpm. The weight of the roadheader is 85t, the cutting power and the total installed power are 260kW and 392kw, respectively. The roadheader can be used for cutting full-rock roadways with the UCS no more than 80MPa. According to the structural design parameters of the roadheader and the roadway conditions, and taking into account the swing and drilling process, the cutting head is designed as a combination of a cylinder, cone, and sphere, with a maximum diameter of 970mm and an axial height of 1020mm, and installed on the drive shaft with one M36 bolt.

#### 3.1.1 The relationship between the helix count and TMR

The cutting heads with 2, 3, and 4 helices are calculated and compared by the same geometric shape for the longitudinal-axis cutting head. The helix angle range of the cylinder, cone sphere are respectively 12–16°, 12–16°, and 12–34°, and the calculation step of helix angle is set to 1°. The range of cutting line spacing between adjacent picks on the same helix is from 48mm to 108mm, and the calculation step of cutting line spacing is set to 0.5mm. In the initial traversal calculation process, a more extensive range is set for each indicator of TMR to obtain all the pick arrangements within the practical design parameter. The range of S_k_ is set from 70° to 115°, the range of S_yP_ and A_TP_ are both 0.7–1.0, and S_yT_ and A_LR_ are set as invalid. The constraint condition is that the decreasing and the variation of the helix spacing are less than 3%. All combinations of cutting line spacing and helix angle are calculated, the results of non-zero traversal TMR with helix count of 2, 3, and 4 are 38313,4650, and 2143 groups respectively. For the cutting head with the same helix spacing, as the helix count decreases, the central angle interval of the picks on the same helix is reduced, which is easy to form the standard mesh, and more non-zero TMR results can be obtained. With the increase of helix count, the central angle interval of the picks on the same helix increases, and the vertex distance of the adjacent and alternate helix relative to vertex P increases, resulting in a decrease in the formation rate of the standard mesh, and the number of non-zero TMR traversal results is reduced.

**Fig 7:**
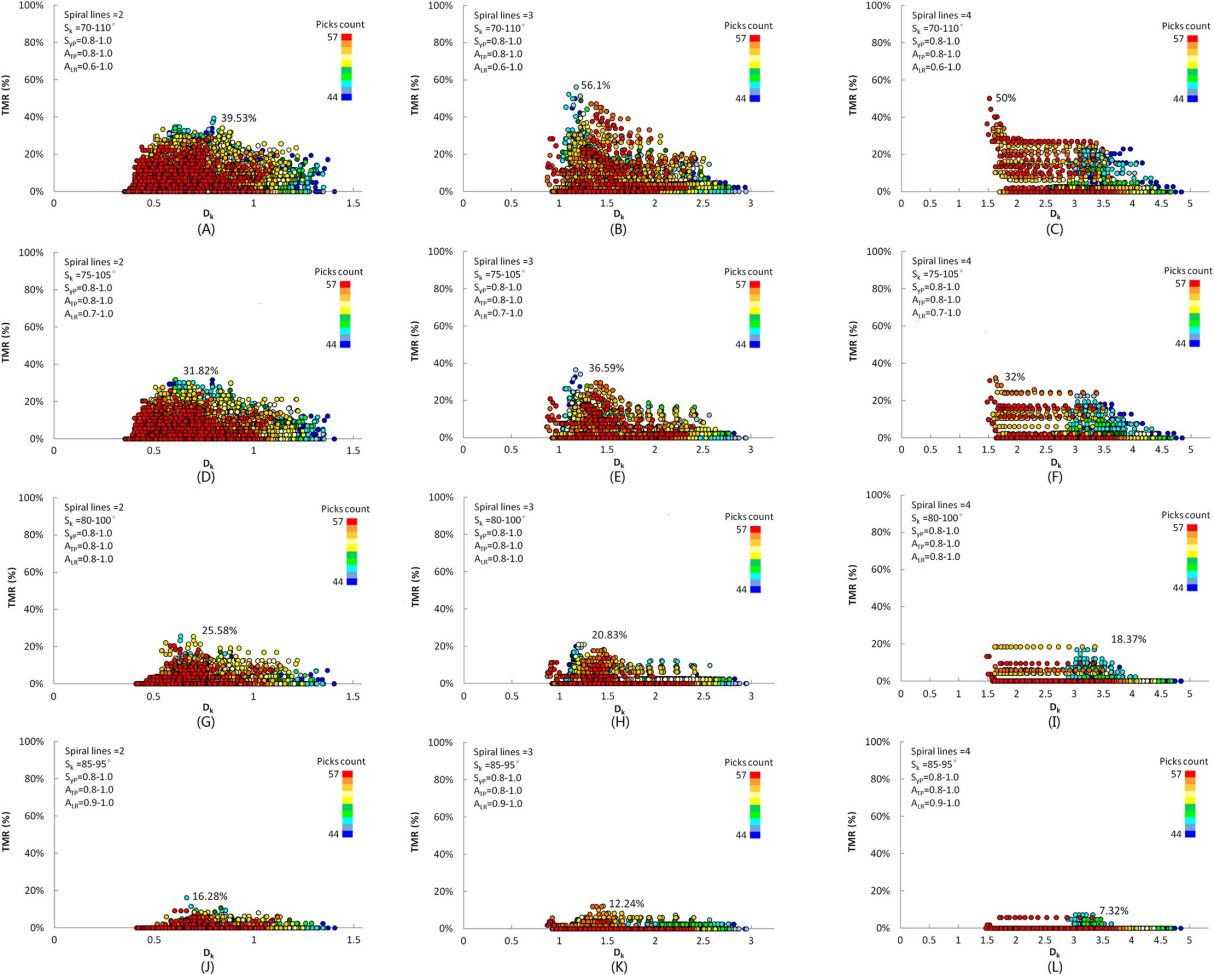
TMR filtering results of cutting head with different helices. (A) 13898 traversal results of 2 helices. (B) 2786 traversal results of 3 helices. (C) 742 traversal results of 4 helices. (D) 11904 traversal results of 2 helices. (E) 1880 traversal results of 3 helices. (F) 512 traversal results of 4 helices. (G) 8100 traversal results of 2 helices. (H) 1038 traversal results of 3 helices. (I) 257 traversal results of 4 helices. (J) 2369 traversal results of 2 helices. (K) 374 traversal results of 3 helices. (L) 66 traversal results of 4 helices.

The traversal calculation results are shown in Fig 7. The abscissa is the ratio of the average distance between pick tips on the same helix to the average distance between adjacent helix for each cutting head expressed by DK. With the improvement of each component of TMR and the reduction of the allowable range of the mesh skewness, the TMR of cutting heads with different helix counts are all reduced, and the count of non-zero traversal results is reduced. For the cutting head with two helices, although the traversal result of the pick arrangement is more than that of the other two kinds of cutting head, the peak value position of the TMR is mainly concentrated in the area of D_k_<1, as shown in Fig 7A, 7D, 7G and 7J. In this case, the spacing between adjacent helix is larger than the average distance between pick tips on the same helix. The accumulated depth of rock cutting groove by picks on the same helix is insufficient to make the rock communicate with each other. Most cut-down rocks are small chips in the form of the cutting groove, which is not suitable for hard rock cutting. However, for the coal cutting with lower UCS, the adequate accumulative cutting groove depth can be obtained, and larger chips between the adjacent helix can be formed. Therefore, the cutting head with 2-helices is more suitable for coal cutting. The TMR of the cutting head with 4-helices in the same constraints is lower for the combination of each component indicator, as shown in Fig 7C, 7F, 7I and 7L. Reducing the helix spacing caused by the increase of helices count will increase the possibility of pick boxes interference at the top of the cutting head, which will result in the inability to establish an effective cutting head model. For the cutting head with 3-helices, the maximum value of TMR appears in the area, where D_k_ is close to 1, and when picks count is more than the average value of the traversal results, the TMR is higher than the other two kinds of cutting head, as shown in Fig 7B, 7E, 7H and 7K.

#### 3.1.2 Variation of the mesh cell area

The mesh cell area decreases with the helix spacing decreases from the bottom to the top of the cutting head. The standard meshes with small area change mainly concentrate on the cylinder and cone, while the standard mesh count is much fewer though the picks are more in the sphere. The decrease of the cutting head diameter, helix spacing, and pick tip spacing causing the skewness angle of the vertex T beyond the set range or small A_TP_ of the mesh cell, which are the main reasons for failing the standard mesh. When the cutting head shape is unchanged and the picks count is the same, the pick distribution density of the cutting head surface is the same. Therefore, the uniformity of the pick arrangement can be analyzed by the changes in the mesh cell area of the cutting head. With the decrease of the coefficient of variation (CV) for the mesh area, each mesh area change becomes less obvious, and the pick arrangement of the cutting head tends to be uniform.

**Fig 8:**
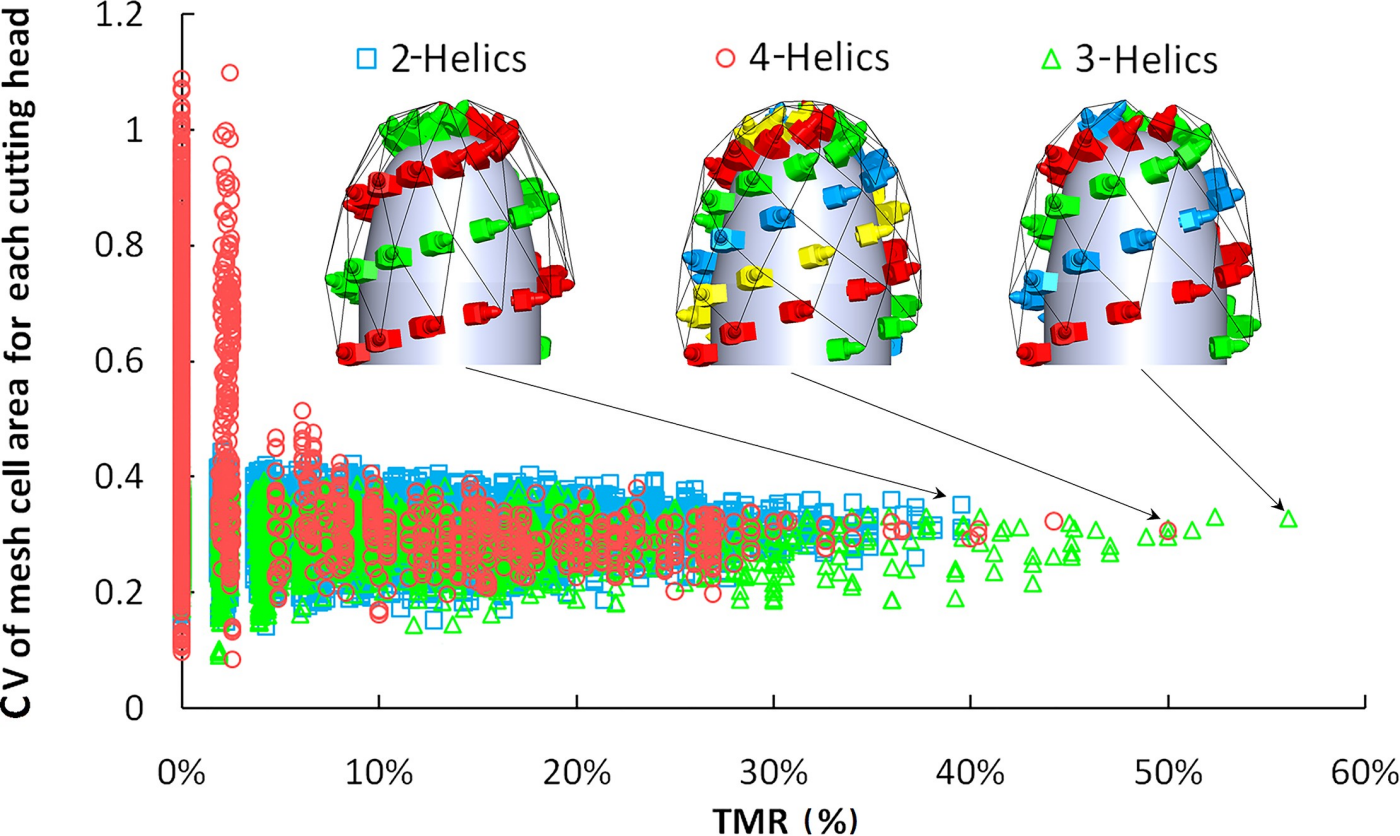
The relationship between CV of mesh cell area and TMR.

The CV results of the mesh cell area for the pick arrangement traversal results of the three kinds of cutting heads are shown in Fig 8. With the increase of TMR, the count of standard mesh increases, the mesh area tends to change continuously, and the corresponding CV decreases significantly. The mesh area uniformity of the 4-helices cutting head is improved most significantly. When TMR reaches the maximum value, the CV of the mesh area for the three kinds of cutting heads are approximately equal, and the TMR of the cutting head with 3-helices is the highest.

#### 3.1.3 Average value of helix spacing and accumulative cutting groove depth

The cutting clip geometry is related to the average helix spacing and the accumulative cutting depth of adjacent picks on the same helix. In rock cutting by a cutting head, the width of cutting clips is affected by the average of helix spacing, denoted by s_s_, and the thickness of the cutting clip is determined by the accumulative cutting depth of adjacent picks on the same helix, represented by d_s_. The length of cutting clips is related to the shape of the helix and motion parameters of the cutting head, and the mechanical properties of rocks. The statistical results of cutting chip size and shape in the laboratory show that the ratio of width to thickness is mostly between 2 to 5 [23, 24]. Similar to the rock cutting by a single pick, when the cutting head design parameters match the operating parameters, the appropriated cutting chips can be obtained with less specific energy consumption.

**Fig 9:**
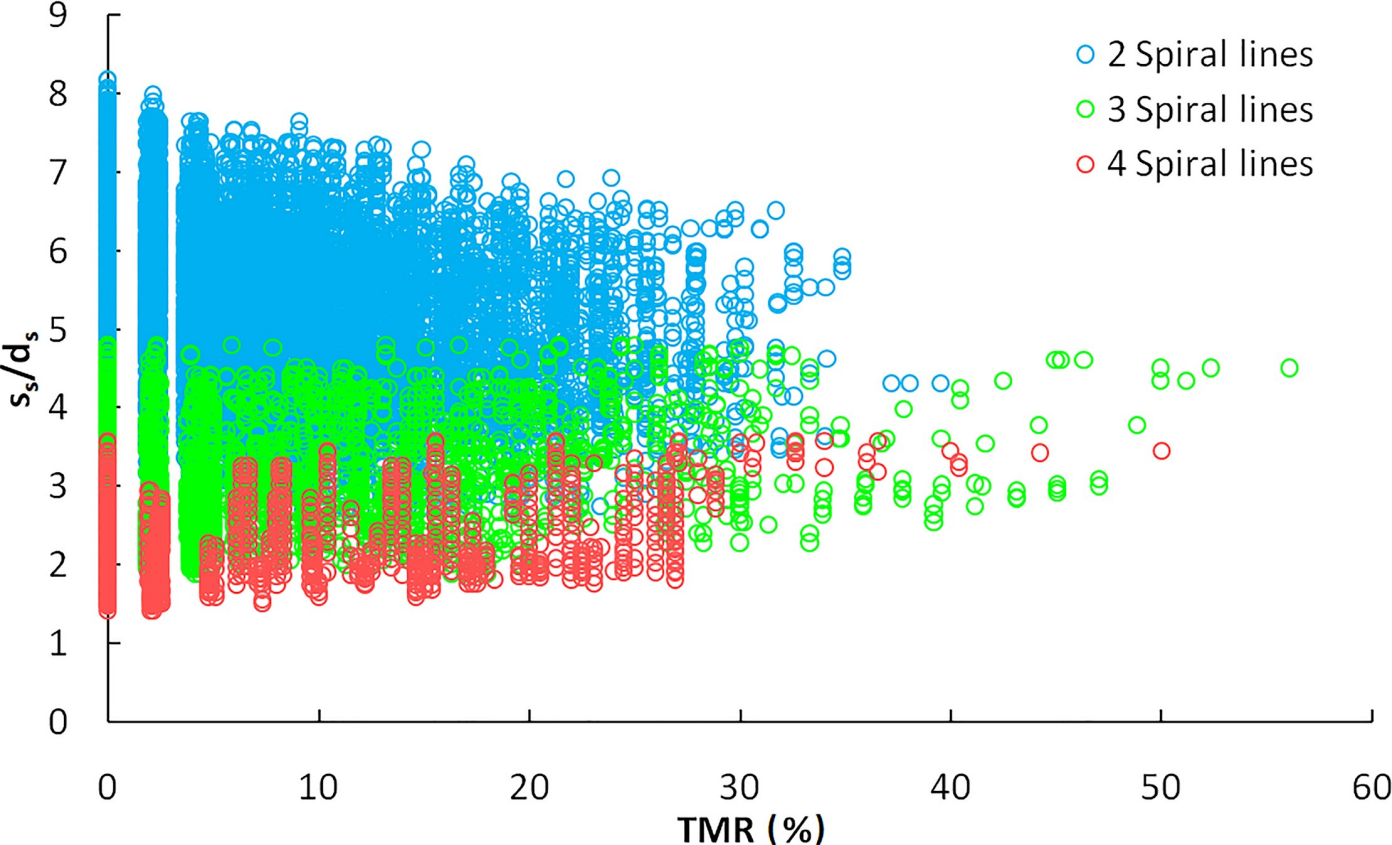
The relationship between TMR and s_s_/d_s_.

As the swing speed and rotation speed of the cutting head change, s_s_/d_s_ changes accordingly. In the conventional rotation speed range of 30-60rpm, for a pick with a shank length of 80mm and attack angle of 45–51°, the maximum cutting depth is 20-35mm without interference between the top of the pick box and rocks, and the corresponding cutting head swing speed range is 0.010–0.035m/s. The average range of s_s_/d_s_ of cutting head with 2-helices, 3-helices, and 4-helices traversal results are 2.7–8.2, 1.9–4.8, and 1.4–3.6. According to the cutting head design parameters of EBZ260W roadheader, when the tungsten carbide at the top of the pick contacts the rock, the average values of s_s_/d_s_ for the three kinds of cutting head are 5.1, 3.0, and 2.2, as shown in Fig 9. The s_s_/d_s_ of 3-helices traversal results is mainly distributed between 2 to 5, which is more beneficial in reducing the specific energy consumption of cutting than the other two kinds of cutting head.

### 3.2 Rock cutting comparison

The cutting head rotates and swings at a certain speed in rock cutting. Each pick penetrates the cross-section passing the cutting head axis in the cutting zone by the sequence of circumference angle and forms a closed cutting chip unit. In the initial process of rock cutting by a pick, the cutting clip unit area is small, so the cutting force is lower. With the continuous rotation and movement of the pick in the cutting zone, the cutting groove gradually deepens. The cutting force on a pick increases with the rise of the chip unit area caused by the deepening of the cutting groove. When the cutting head rotates out of the cutting zone, the cutting clip unit area and the cutting force quickly decrease to zero. Therefore, the cutting pattern of each traversal result of pick arrangements is different. The cutting chip unit area and shape on different cutting sections for the same cutting head will be different, further affecting the variation of the cutting load composed by the cutting force of each pick along the swing direction and vertical direction.

#### 3.2.1 Pick rotation coefficient

In any cutting section, the cutting chip unit consists of vertex P_P_, the left and right crack boundary vertex P_L_ and P_R_, and the adjacent cutting chip unit boundary or cutting head contour. Each cutting chip unit in the section forms a cutting pattern according to the sequence of pick rotation into the section. The cutting section parallels the swing direction and with the largest cutting depth can be defined as the main cutting section and represented by π_0_. The vertex P_P_ of each chip unit is determined by the rotation speed and swing speed of the cutting head, and the projection line of the pick axis through the vertex P_P_ on the section divides the cutting chip unit into left and right sides. The shape of each side is determined by the distance between adjacent picks, rock broken angle, and the boundary of the adjacent cutting groove determined by the accumulative cutting depth. In addition, the difference in accumulative cutting depth caused by the difference in linear speed of picks on the cylinder and sphere in the swing process will also change the length of the crack boundary.

**Fig 10:**
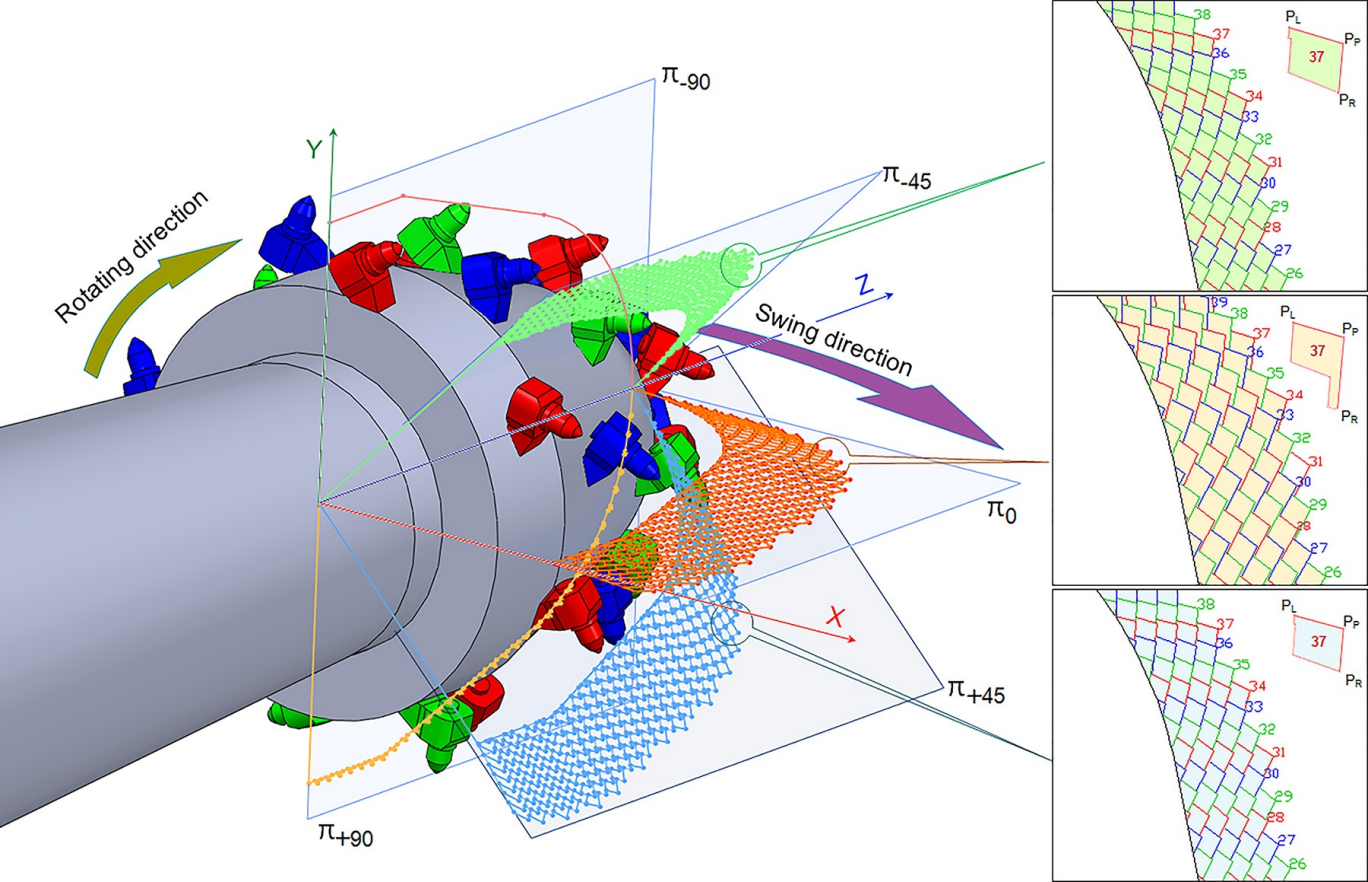
Pick rotation coefficient on different cutting sections.


The cutting chip unit can usually form with a hexagonal geometry by the uniform pick arrangement and a proper conical shank length. The cutting clip unit only contains another chip unit vertex except for the current pick, as shown in Fig 10. Since the longitudinal-axis cutting head usually uses a right-handed helix to arrange the picks, the right side of the cutting chip unit for each pick is longer than that of left side. Therefore, the right to left crack boundary ratio is defined as the pick rotation coefficient K_r_, see Eq (12). In the effective cutting zone of 180°, as the cutting head rotates, the depth of a single-pick cutting groove always increases from 0 to the maximum value and then decreases to 0. The pick constantly changes the cutting state by relieved cutting mode, unrelieved cutting mode, or transitional cutting mode, so K_r_ of the same pick in different cutting sections will vary with the shape of the cutting chip unit. For instance, K_r_ of the No.37 pick, in π_-45_ cutting section, is 0.86, while in π_0_ cutting section, K_r_ increases to 1.33, and in π_+45_ cutting section, it decreases to 0.86. When K_r_>1, due to the right boundary has a larger contact length with the rock than that of the left side, the friction torque on the right side is greater, resulting in the pick rotates clockwise viewing from pick tip to body. In contrast, the pick rotates counterclockwise while K_r_<1.

$$Kr=\frac{P_{P}P_{R}}{P_{P}P_{L}}$$ (12)

**Fig 11:**
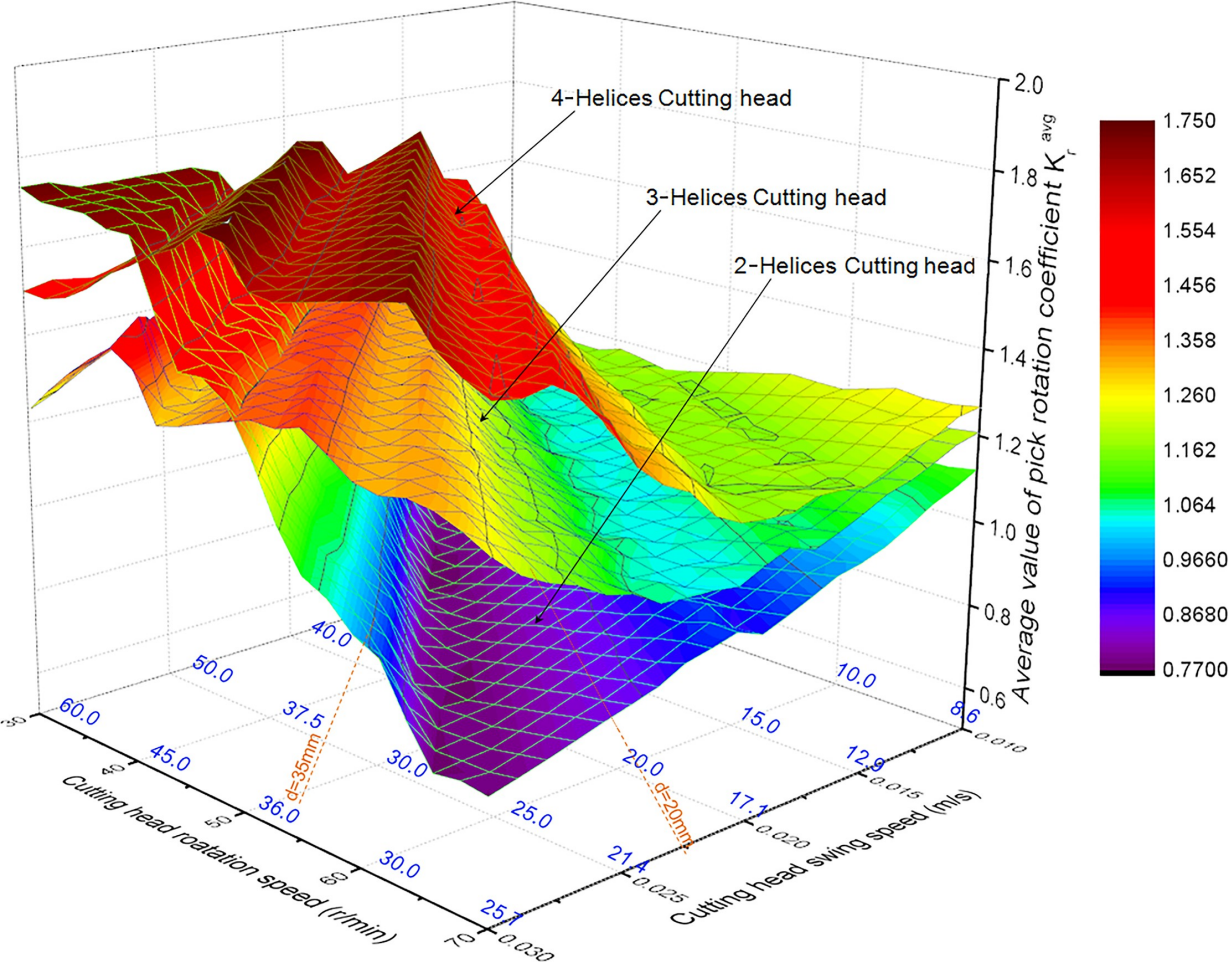
The influence of swing speed and rotation speed on the average value of pick rotation coefficient.

Within the range of roadheader working parameters, the pick arrangement corresponding to the maximum TMR of the three kinds of cutting heads with different helices was calculated. The average value of rotation coefficient K_r_^avg^ in section π_0_ with the maximal cutting depth is shown in Fig 11. The intersection point of the swing and rotation speed in the figure is the average cutting depth of each pick, signed by*d*. Taking the pick box is in non-contact with the rock as the constraint condition, the calculation result of the cutting depth is 20-35mm. The variation range for the average of pick rotation coefficient corresponding to the 3-helices cutting head is minimum and the K_r_ is mainly in the range of 1.05–1.40. When the rotation speed of the cutting head remains unchanged, each pick has a similar rotation effect; the swing speed of the cutting head is relatively more extensive. The K_r_ range of the 2-helices cutting head is 0.75–1.23. When the cutting head rotates at a constant speed, the pick rotates in clockwise and anticlockwise rotation alternately in the pick box. As the cutting head swing speed increases, picks are prone to wear on both sides. For 4-helices cutting head, the K_r_ range is 1.22–1.71, and the pick rotates in clockwise rotation significantly, which is easy to cause rapid wear on one side of the conical shank.

#### 3.2.2 Cutting load variation

The cutting load of the roadheader includes the cutting force of each pick during the rock cutting process. Due to the difference of pick arrangement, the sequence and time interval of picks cutting into the rock at the same time are different, causing the cutting load to be various. When the pick arrangement is uniform, the variation of the cutting load is slight, and the cutting load is stable. Set an XYZ coordinate system along the swing direction of the cutting head. The X-direction is consistent with the swing direction, and the Z-direction is consistent with the axis of the cutting head and points to the top of the cutting head. The cutting load variation can be analyzed by the CV of each component in X, Y, and Z directions. In the cutting simulation, the cutting force, normal force, and side force on a pick of relieved or unrelieved cutting state scan be calculated according to N.Bilgin’s single pick cutting force model based on rock cutting tests of various UCS [25]. The combined cutting load for the cutting head based on picks in the cutting zone can be calculated by Eq(13) to Eq (16).


$$F_{X}=\sum_{\omega =0}^{2\pi k}\sum_{i=1}^{m}\left(F_{C_{i}}^{N.Bilgin}(\omega )\sin\omega -F_{N_{i}}^{N.Bilgin}(\omega )\cos\omega \cos\beta_{i}\right)$$ (13)

$$F_{Y}=\sum_{\omega =0}^{2\pi k}\sum_{i=1}^{m}\left(F_{C_{i}}^{N.Bilgin}(\omega )\cos\omega +F_{N_{i}}^{N.Bilgin}(\omega )\sin\omega \cos\beta_{i}\right)$$ (14)

$$F_{z}=\sum_{\omega =0}^{2\pi k}\sum_{i=1}^{m}(F_{S}{}_{i}(\omega )+F_{N_{i}}^{N.Bilgin}(\omega )\sin\beta_{i})$$ (15)

$$P=\frac{\sum_{i=1}^{m}(F_{C_{i}}^{N.Bilgin}\times Rg_{i})\times n}{9550}$$ (16)

Where *F*_*C*_^*N*. *Bilgin*^ is the cutting force of a single pick opposite to the rotation direction of the cutting head, kN. *F*_*N*_^*N*. *Bilgin*^ is the normal force of a single pick pointing to the axis of the cutting head, kN; and *F*_*s*_ is the side force from *F*_*N*_^*N*. *Bilgin*^ to *F*_*C*_^*N*. *Bilgin*^ with the right hand, kN. *P* is the cutting power, kW. *ω* is the circular angle of cutting head at the current simulation time, rad. *k* is cutting head rotation times. *i* is the serial number of current pick. *m* is the picks count in cutting zone. n is the rotation speed of the cutting head, r/min. The rest symbols are the same as before.

**Fig 12:**
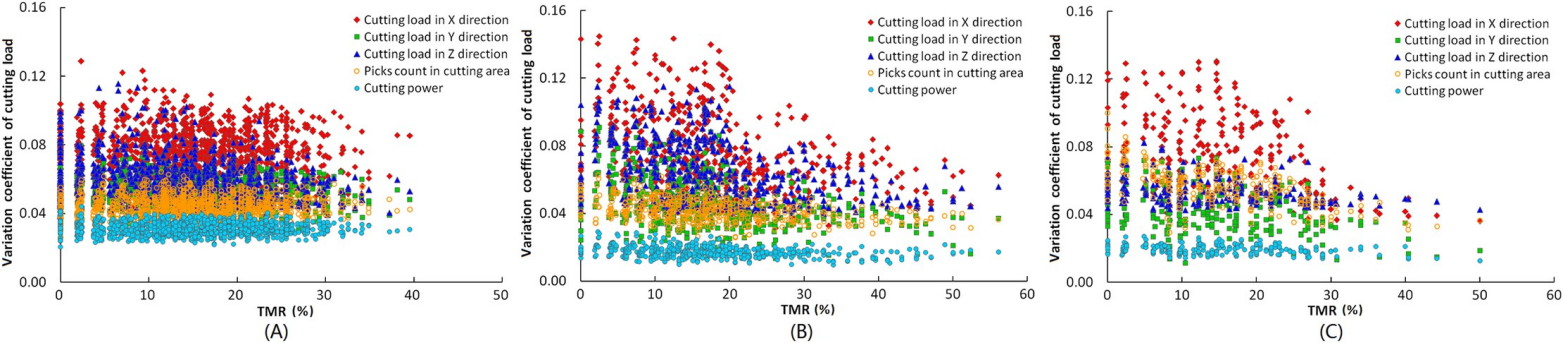
Relationship between CV of cutting load and TMR of each cutting head. (A) 2-helices cutting head. (B) 3-helices cutting head. (C) 4-helices cutting head.

For reducing the calculation scale without reducing the representativeness of the calculation results, all the traversal results for each helical number are grouped by picks count, and the traversal results with different TMR are extracted from the same picks count groups for simulation of the cutting process. 1260, 521, and 245 traversal results can be obtained with 2, 3, and 4 helices, respectively. For the conventional rock roadway with an average UCS of 60Mpa, as the cutting head swings, the cutting depth of each pick increases from 27mm to 34mm with the direction of the bottom to top of the cutting head. The relationship between the CV of cutting load and TMR with the rotation speed of 32.5rpm is shown in Fig 12. As the TMR increases, the uniformity of pick arrangement is improved. The CV of cutting load in XYZ direction of the cutting head with 2, 3, and 4 helices is significantly reduced, and the cutting load stability is gradually improved. However, the CV of cutting power has no significant change with the same conditions, which indicates that for the same helix numbers of the cutting head, the correlation between the cutting power variation and the pick arrangement parameters is low.

### 3.3 Cutting head design with 3-helices

According to the above analysis results, to obtain a uniform pick arrangement, reduce the cutting load variation during rock cutting, and improve the adaptability of the cutting head at different swing speeds, the cutting head of EBZ260W roadheader adopts 3-helices for pick arrangement.

#### 3.3.1 Design comparison with different picks count

In traversal results of 3-helices cutting head, the TMR indicators are further filtered to obtain more uniform pick arrangement parameters. The range of S_K_ is reduced to 90–110°; S_YP_ and S_yT_ are reset to 0.8–1.0 and 0.7–1.0; A_TP_ and A_LR_ are reset 0.8–1.0 and 0.6–1.0, respectively. The helix angle and cutting line spacing are shown in Table 1. The 11 results for pick arrangement with the largest TMR among different picks count are selected for 3D modeling of the cutting head, as shown in Table 2. After correcting the pick position without interference on top of the cutting head, the new results of pick count and TMR recalculated are shown in Table 3.

**Table 1:** Helix angle and cutting line spacing of 3-helices cutting head.

Cutting head	Helix angle of the cylinder (°)		Helix angle of the cone (°)		Helix angle of the sphere (°)		cutting line spacing (mm)
	starting value	end value	starting value	end value	starting value	end value	
CH42A	15	15	16	16	12	28	81.0
CH42B	15	15	16	16	12	28	79.5
CH44	15	15	16	16	12	28	76.5
CH45	14	14	15	15	12	25	75.0
CH46	14	14	15	15	12	25	73.5
CH48	13	13	14	14	12	24	70.5
CH49	13	13	14	14	12	23	69.0
CH50	13	13	14	14	12	23	67.5
CH51	12	12	13	13	13	21	66.0
CH52	12	12	13	13	13	19	64.5
CH53	12	12	13	13	14	17	63.0

**Table 2:** Initial traversal results.

Traversal results	Cutting head										
	CH42A	CH42B	CH44	CH45	CH46	CH48	CH49	CH50	CH51	CH52	CH53
Picks count	44	45	46	47	49	50	52	53	54	55	57
Pick arrangement mesh count	31	33	33	34	35	37	39	39	39	41	42
Standard mesh count	20	23	14	19	22	17	20	18	22	22	18
D_K_	1.16	1.17	1.09	1.29	1.22	1.28	1.25	1.25	1.43	1.49	1.65
TMR (%)	50.00	56.10	33.33	44.19	48.89	36.96	41.67	36.73	44.00	43.14	33.96

**Table 3:** Recalculated results without interference.

Traversal results	Cutting head										
	CH42A	CH42B	CH44	CH45	CH46	CH48	CH49	CH50	CH51	CH52	CH53
Picks count	42	42	44	45	46	48	49	50	51	52	53
Pick arrangement mesh count	30	30	32	32	34	35	37	37	39	40	42
Standard mesh count	17	19	14	19	20	17	20	18	25	22	18
D_K_	1.14	1.15	1.06	1.28	1.20	1.27	1.225	1.22	1.40	1.47	1.63
TMR (%)	44.74	50.00	35.00	46.34	47.62	38.63	44.44	39.13	53.19	45.83	36.73

The TMR of CH42B and CH51 pick arrangement for the cutting head are more than 50%; however, the helix angle and the distance between the adjacent helices of CH51 are all smaller. For hard rock cutting, since it is difficult to cut into the rock, the CH51 cutting head can still obtain s_s_ / d_S_ in the range of 2–5 with a small cumulative cutting depth.

#### 3.3.2 Cutting load coefficient of variation for different cutting thickness

**Fig 13:**
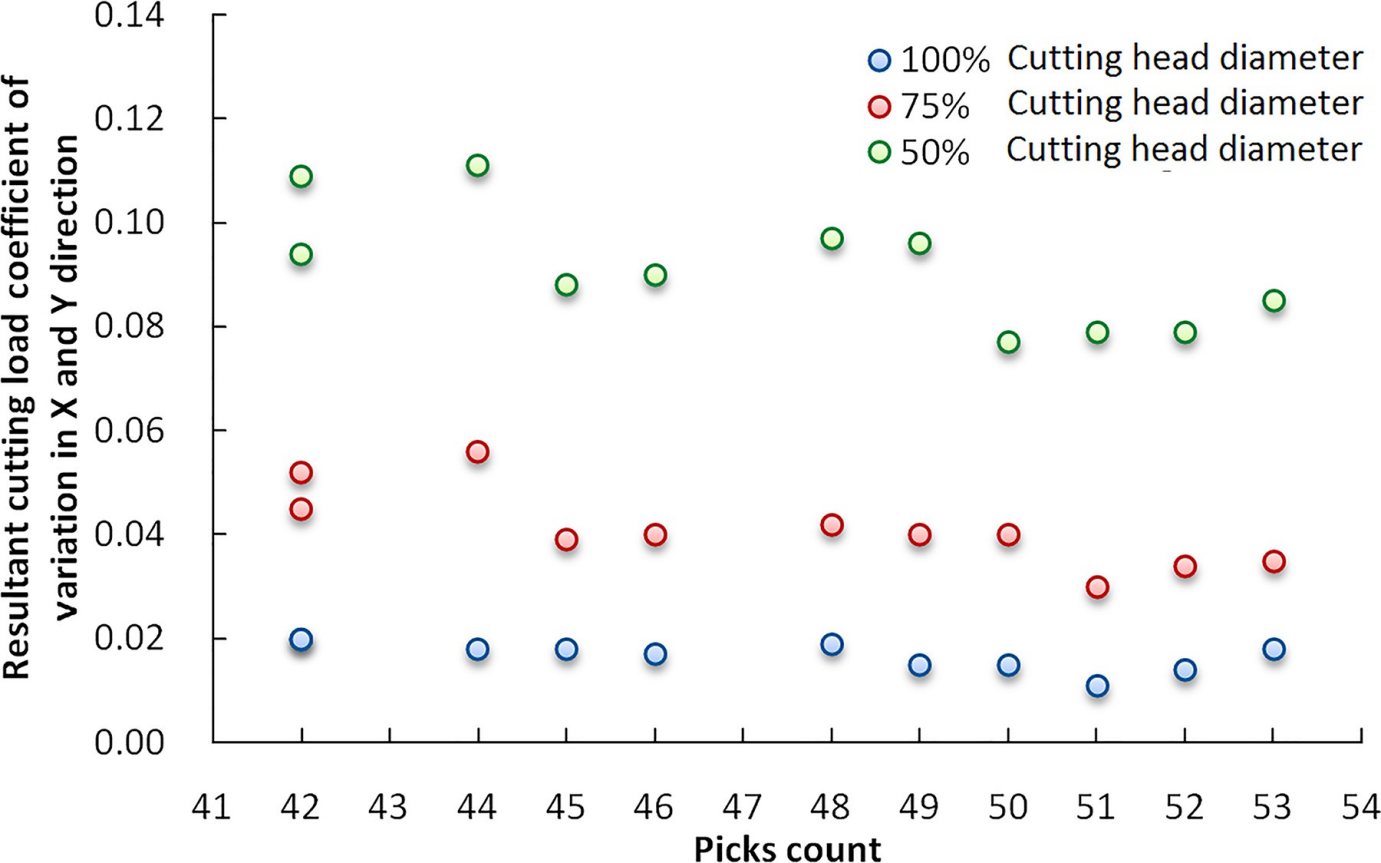
Cutting load CV and cutting thickness.

To analyze the cutting load variation of each cutting head with different cutting thickness, set the UCS for rock cutting simulation from 60 to 80MPa; and the whole cutting head is drilled into the rock. The cutting thickness is set to 100%, 75%, and 50% of the cutting head diameter, respectively. The cutting load CV of resultant force in X and Y direction for 11 kinds of cutting heads are shown in Fig 13. The variation of cutting load decreases with the increase of cutting thickness; however, better working efficiency can be obtained with the cutting thickness usually beyond 75% of the cutting head diameter. Therefore, the resultant cutting load CV of the CH51 pick arrangement is the smallest for different cutting thicknesses, and the cutting load stability is higher than the other ten results.

#### 3.3.3 Determination of cutting head design parameters

**Fig 14:**
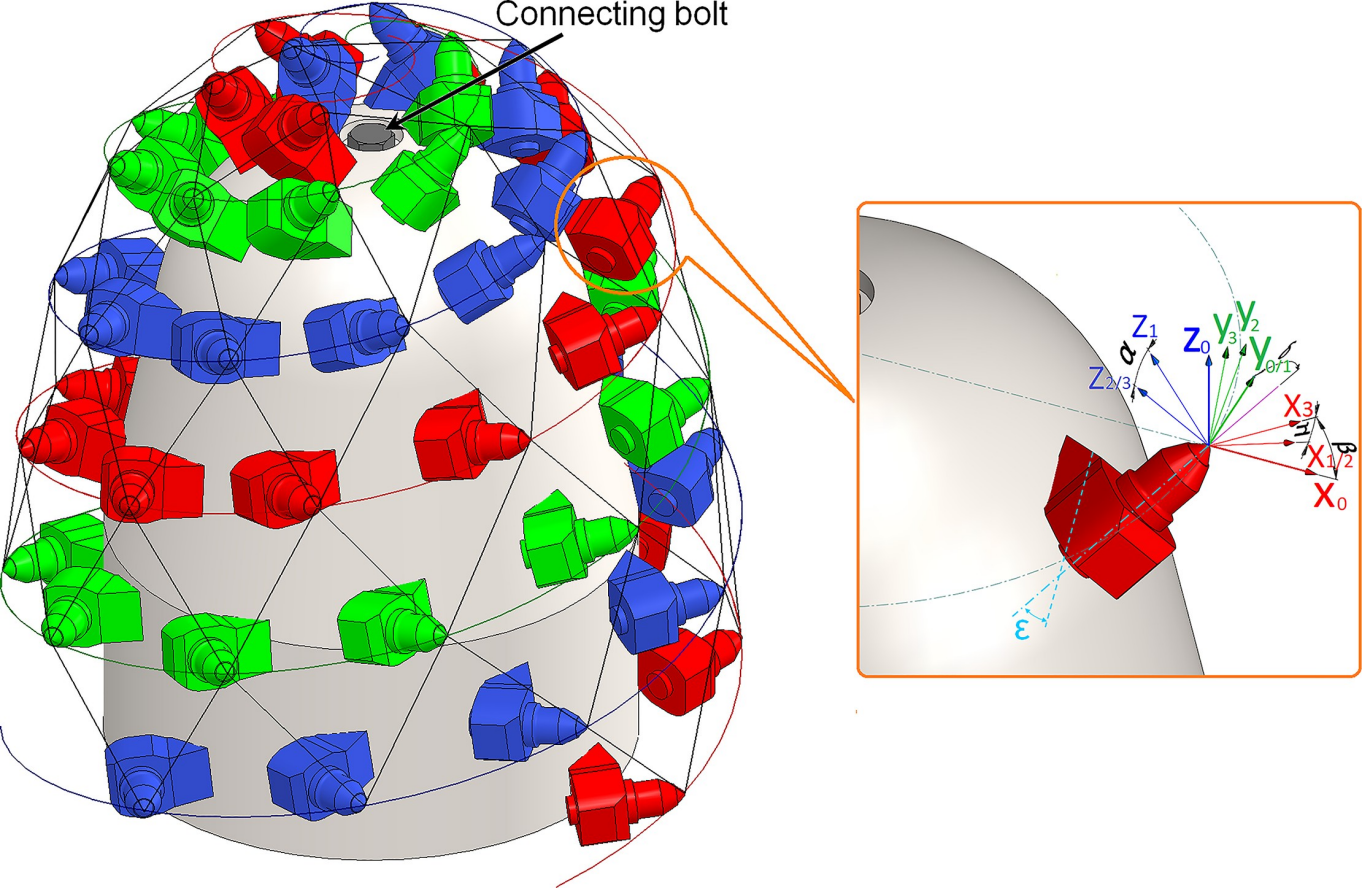
3D model of CH51 cutting head.

**Fig 15:**
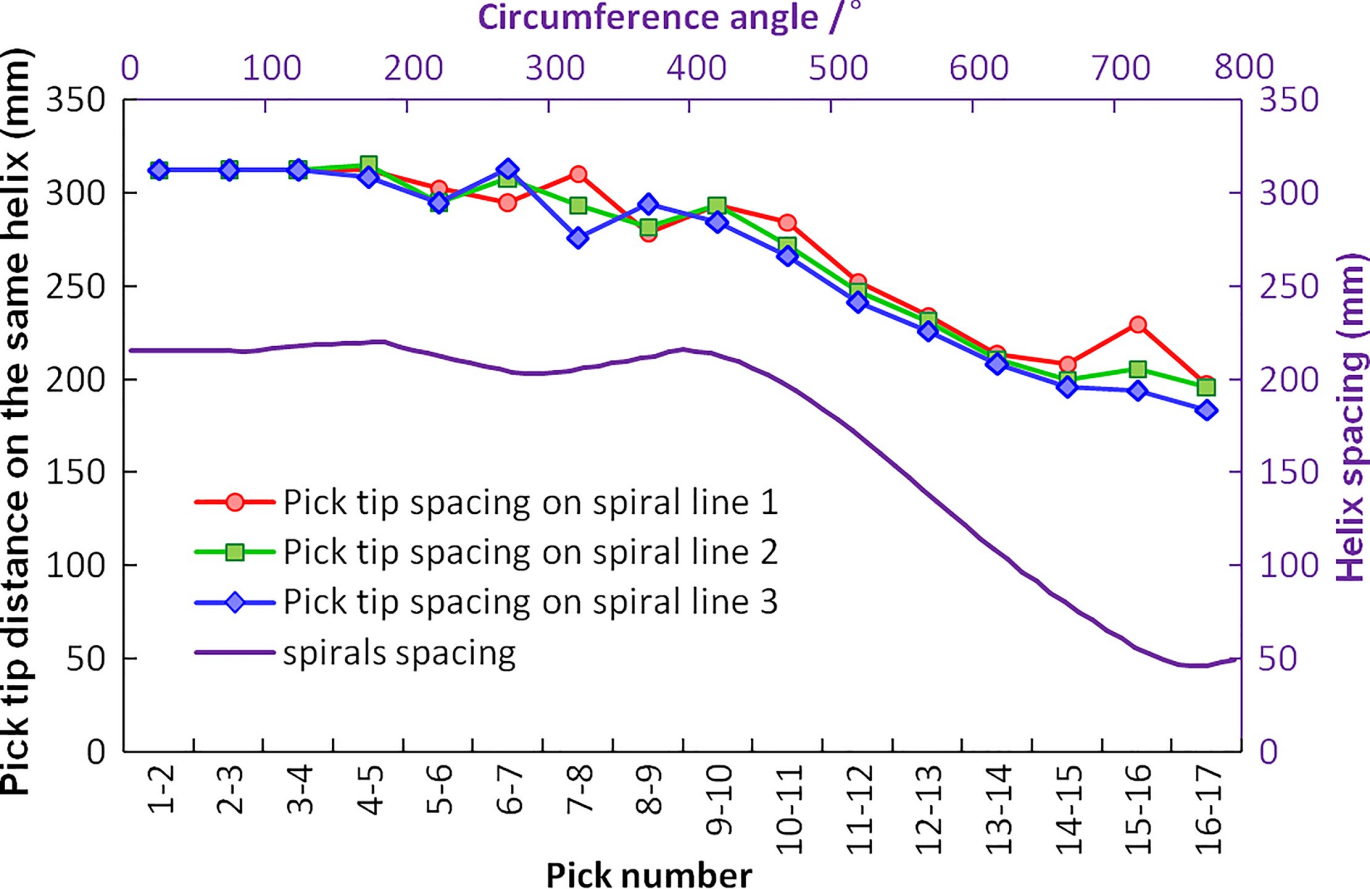
The adjacent helix spacing and the adjacent pick tip distance of CH51 cutting head.

Based on the above analysis, the cutting head is designed with CH51 pick arrangement parameters, and the 3D model of the CH51 cutting head is shown in Fig 14. For a significant cutting effect, the attack angle δ is determined by tilt angle, skew angle, and elevation angle and set from 49°to 50°. X_0_ axis of the initial coordinate X_0_Y_0_Z_0_ of the pick positioning process is perpendicular to the cutting head axis, and the Z_0_ axis is consistent with the cutting head axis. The tilt angle, expressed by β, is the rotation angle of the coordinate X_1_Y_1_Z_1_ around the Y0 axis and is used to make the bottom surface of the pick box and the surface of the cutting head fully fit. Its value varies with the curvature of the cutting head with the range from 0° to 90°. The tilt angle is 0°on the cylinder and gradually increases from 0° to 11.4° of the three picks transitioning from the cylinder to the cone. On the sphere, the tilt angles gradually increase from 13.8° to 75.8°. The skew angle is the rotation angle of the coordinate X_2_Y_2_Z_2_ around the X_1_ axis. It can balance the torque generated by the friction on both sides of the pick axis and makes the pick obtain a good rotation effect in the pick box, represented by*α*. The skew angle of the picks on the cylinder and cone is set at 8°.For assembling the connecting bolt, the pick skew angles at the end of each helix are reversely rotated from 8° to -9°. The elevation angle is the rotation angle of the coordinate X_3_Y_3_Z_3_ around the Z_2_ axis and can be represented by μ, and the attack angle for rock cutting can be determined by elevation angle and pick box mounting angle *ε*. For a pick box with an installation angle of 45°, the preset attack angle can be reached with the elevation angle from 3.53° to 4.53°. The average value of helix spacing between the cylinder and the cone is 213mm, the cutting line spacing on the same helix is 66mm, and the average distance between adjacent picks is 299mm. As a result, the corresponding D_k_ is 1.4. On top of the sphere, the helix spacing and the pick tip distance decrease with the reduction of the cutting line spacing, as shown in Fig 15. In rock cutting simulation, only the tungsten carbide interacts with the rock mass, so the cutting depth is usually 5mm to 10 mm, and the range of s_s_/d_s_ is from 2.1 to 4.3.

**Fig 16:**
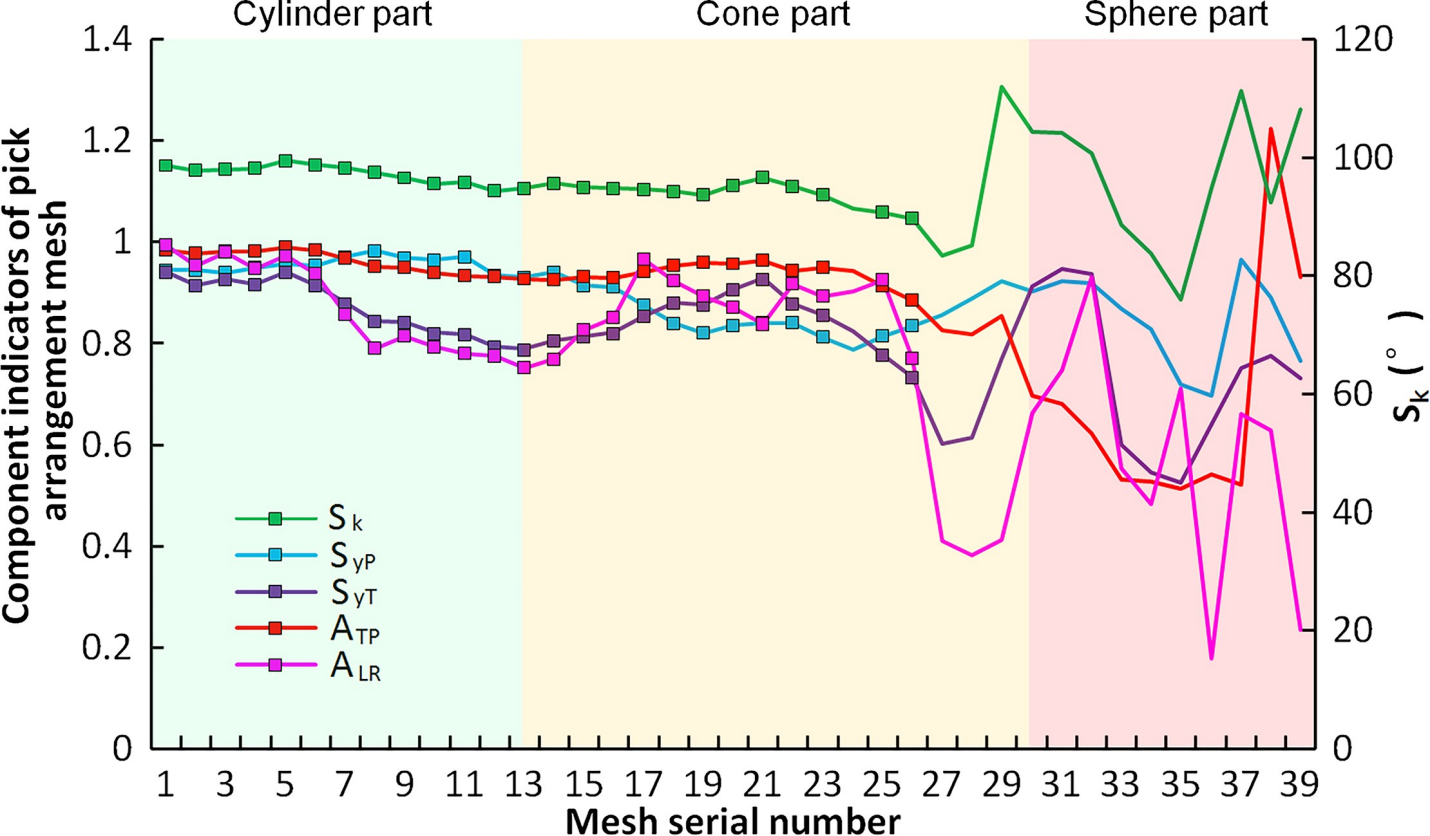
TMR indicators of the CH51 cutting head.

For CH51 cutting head with the TMR of 53.19%, each indicator is shown in Fig 16, where the marked points symbolize the standard meshes. The standard meshes are mainly concentrated on the cylinder and the cone, while the sphere can hardly form any standard mesh. This phenomenon is caused by the decrease in the alternate helix spacing of the transition area from the cone to the sphere, resulting in a reduction in the area ratio of upper to lower triangular elements, which cannot meet the standard mesh forming conditions. In addition, the vertex L and R positions of the mesh element on the cone remain unchanged, while the significant change in helix angle of the sphere causes a large offset in the circumferential position of vertex T, result in a considerable change in mesh area ratio of the left to right triangular elements, which is another main reason for unable to form the standard mesh. In the swing of rock cutting, the picks on the cylinder and cone are the primary cutting tools, and the cutting volume is more considerable; although there are more picks on the sphere, the cutting volume of each pick is small, and the direct cutting force on rock is lower. Therefore, the mesh ratio of the sphere has no significant influence on cutting load variation.

### 3.4 Rock cutting simulation of CH51 cutting head

Pick consumption and rock cutting specific energy are important indicators to evaluate the design reasonableness of cutting head. Among them, pick consumption is a directly observable index, which can be used to measure the design performance of the cutting head; the specific energy is used to analyze the cutting performance of the cutting head or the working efficiency of the roadheader.

#### 3.4.1 Pick consumption analysis

Pick consumption is related to various factors [26], such as rock characteristics and pick material, shape and size of the cutting head, pick arrangement and spatial attitude, cutting speed and cutting depth, the output torque of the cutting head, continuous working time, and so on. Due to the complex working conditions of roadheader, there is no obvious correlation between the factors for the pick consumption. Therefore, this paper uses a qualitative method to analyze pick consumption.

When the production quality of picks is stable and the rock properties change insignificantly, the pick consumption on the cutting head is mainly related to the CERCHAR abrasivity index (CAI), directly rock cutting volume, cutting speed and the rotation coefficient. The CAI can be obtained by the pin wear test and determined by diameter section wear caused by the movement of the pin tip on the rock. The abrasiveness of the rock can be divided into seven grades from extremely low to extremely high, and CAI is positively correlated with the pick wear [27, 28].The directly cutting volume by a pick is determined by geometry of the cutting groove, and the cutting groove area increases with the cutting depth. Under the combined action of skew angle and elevation angle, the cutting groove asymmetry of the left and right sides increases, and the pick rotation coefficient changes significantly. With the action of unbalanced friction torque, the risk of pick wears increases. The cutting speed is determined by the combination of the cutting head rotation speed and the swing speed. Since the pick is distributed along the axis of the cutting boom, the cutting speed of each pick increases with the increase of the distance from the swing center to the pick tip. The rock cutting test show that the pick wear consistent with the cutting speed [29].

**Fig 17:**
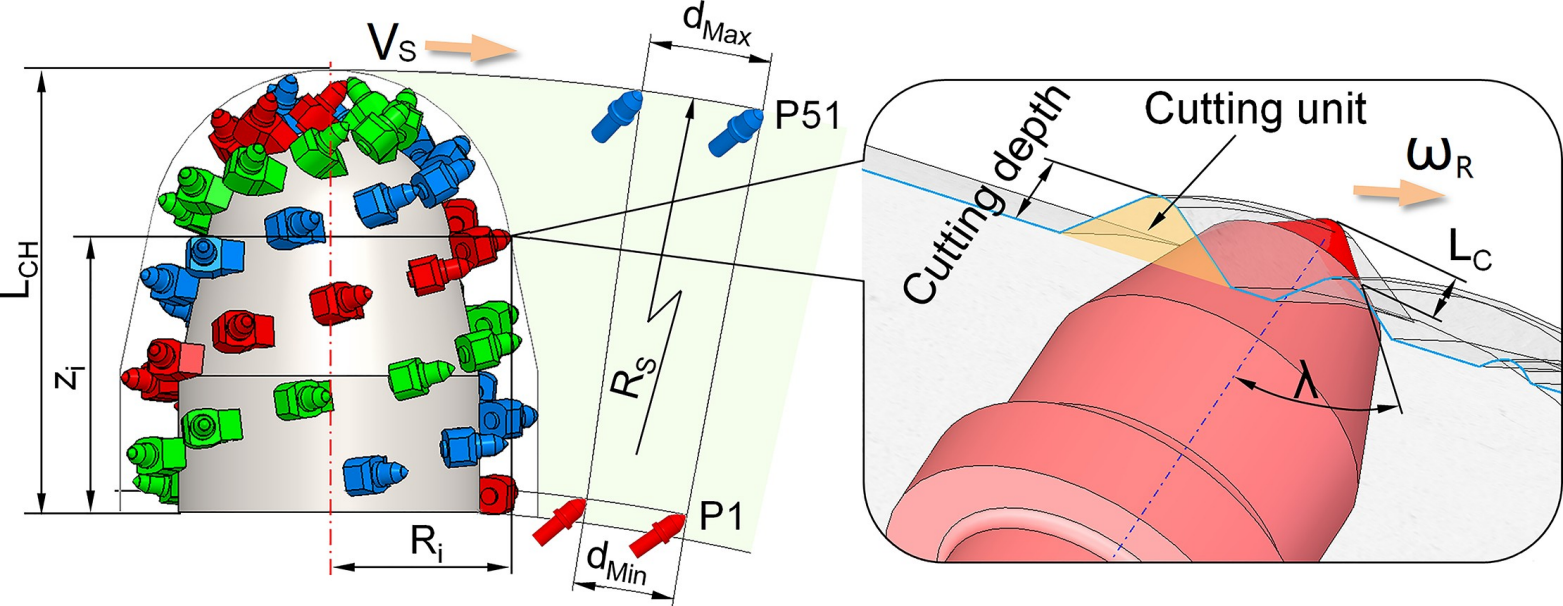
Rock cutting model with a longitudinal cutting head.

The cutting model of the CH51 cutting head is shown in Fig 17. The axial distance from the first pick P1 to the last pick P51 is 970mm. Therefore, the cutting depth is different in the swing cutting of each pick. For a conventional pick with the half cone angle λ of the tungsten carbide is from 40° to 60; the effective height L_C_ of tungsten carbide is 15-18mm, and the attack angle δ is 45–55°. With the pick box is not in contact with the rock as the restriction condition, the maximum cutting depth d can reach 33mm with the conical shank of the pick cutting into the rock, and the corresponding swing speed v_S_ is 0.018m/s. Divide the speed value equally; the cutting depths of P1 and P51 on the π_90_ section with the largest cutting depth are shown in Table 4.

**Table 4:** Cutting depth with various cutting head moving speed.

Cutting depth of a rotation (mm)	Cutting head swing speed (m/s)								
	0.002	0.004	0.006	0.008	0.010	0.012	0.014	0.016	0.018
d_min_	3.0	6.0	8.9	11.9	14.9	17.9	20.8	23.8	26.8
d_max_	3.7	7.4	11.0	14.7	18.4	22.1	25.8	29.5	33.1

**Fig 18:**
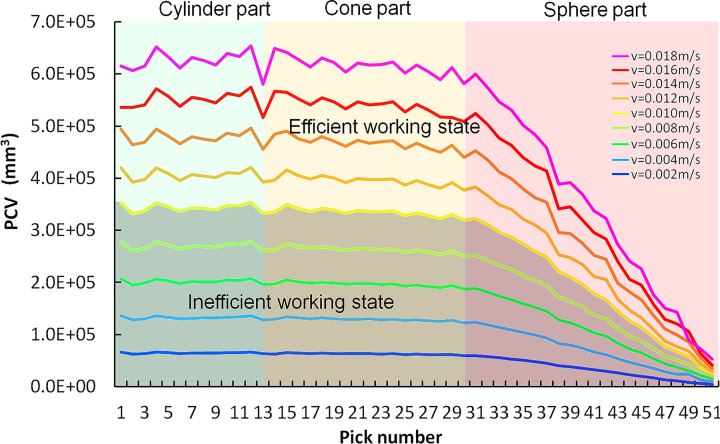
Cutting volume of each pick for one rotation.

The direct rock cutting volume of each pick (PCV) by rotating in a circle at different swing speeds is shown in Fig 18. With the increase of swing speed, the PCV increases. Due to the interaction between pick and rock, the height loss and mass loss of picks on the cutting head should be consistent with the changing trend of cutting volume. For the rocks with low UCS and CAI, the cutting depth of each pick is considerable, and the cutting head swings at a higher speed, which is an efficient cutting state. For high UCS and CAI rock cutting, it is difficult for the pick to cut into the rock, and the cutting head is in an inefficient working state with low swing speed. With the increase of the pick swing radius, the cutting depth of P51 is 23–24% more than that of P1, and the actual rock cutting volume is significantly reduced by 91.4–93.2%. Therefore, the picks on top of the cutting head are in an inefficient working state, and the height loss of tungsten carbide at the top of the pick will not increase significantly with the increase of rock cutting depth for the swing.

**Fig 19:**
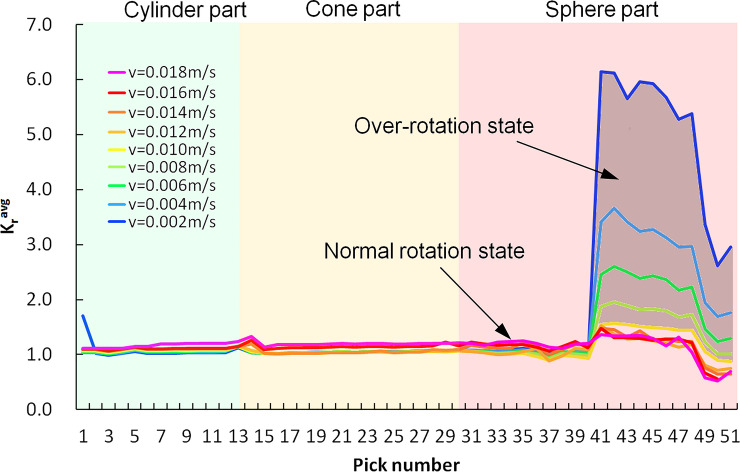
Rotation coefficient of each pick.

During the swing of the rotating cutting head, when the cutting speed is different, various geometry of cutting chips will change the average value of pick rotation coefficient in the cutting area, as shown in Fig 19. For various swing speeds, the K_r_^avg^ of the picks on the cylinder, cone, and its adjacent part of the sphere on the cutting head varies from 0.99 to 1.32, and each pick is in a normal rotation state. The pick on the sphere is affected by the gradual increase of the skew angle. From P41, the left side of the cutting chip scan no longer intersect with the cutting clip unit formed by adjacent picks. It can only intersect with the right side of the same cutting unit formed in the last rotation, which results in a significant increase in K_r_^avg^. When the cutting head moves at a low swing speed, only the tungsten carbide part of the pick cuts into the rock, and the conical shank part of the pick does not contact the rock, so the risk of pick wear is still low. As the swing speed increase, the cutting depth increase, and the asymmetry of the left and right cutting groove section makes the pick over-rotate on the side with a considerable contact length with the rock, which may cause conical shank wear and mass loss. For the swing speed beyond 0.010m/s cases, the K_r_^avg^ of P41 to P51 deviated from the normal rotation state by +24.2% to -33.2%, respectively. Therefore, the risk of mass loss of these picks is higher than that of picks on other positions on the cutting head.

The cutting speed is the vector sum of the pick rotating linear speed around the cutting head axis and the swinging linear speed of the cutting arm. It is related to the rotation radius of the pick to the cutting head axis and the distance from the pick tip to the swing center and can be calculated by Eq(17).


$$v_{\Sigma }=\frac{R_{S}-(L_{CH}-Z_{i})}{R_{S}}v_{S}+\frac{\pi n}{30}R_{i}$$ (17)

Where *V*_*Σ*_ is the cutting speed, m/s.*R*_*S*_ is the swing radius of cutting head, mm. *Z*_*i*_ is the axial position of any pick on the cutting head. *R*_i_ is the rotation radius of the pick around the cutting head axis, mm. *L*_*CH*_ is the length of the cutting head, mm. *v*_*S*_ is the swing speed of cutting head, m/s. The rest of the symbols are the same as before.

**Fig 20:**
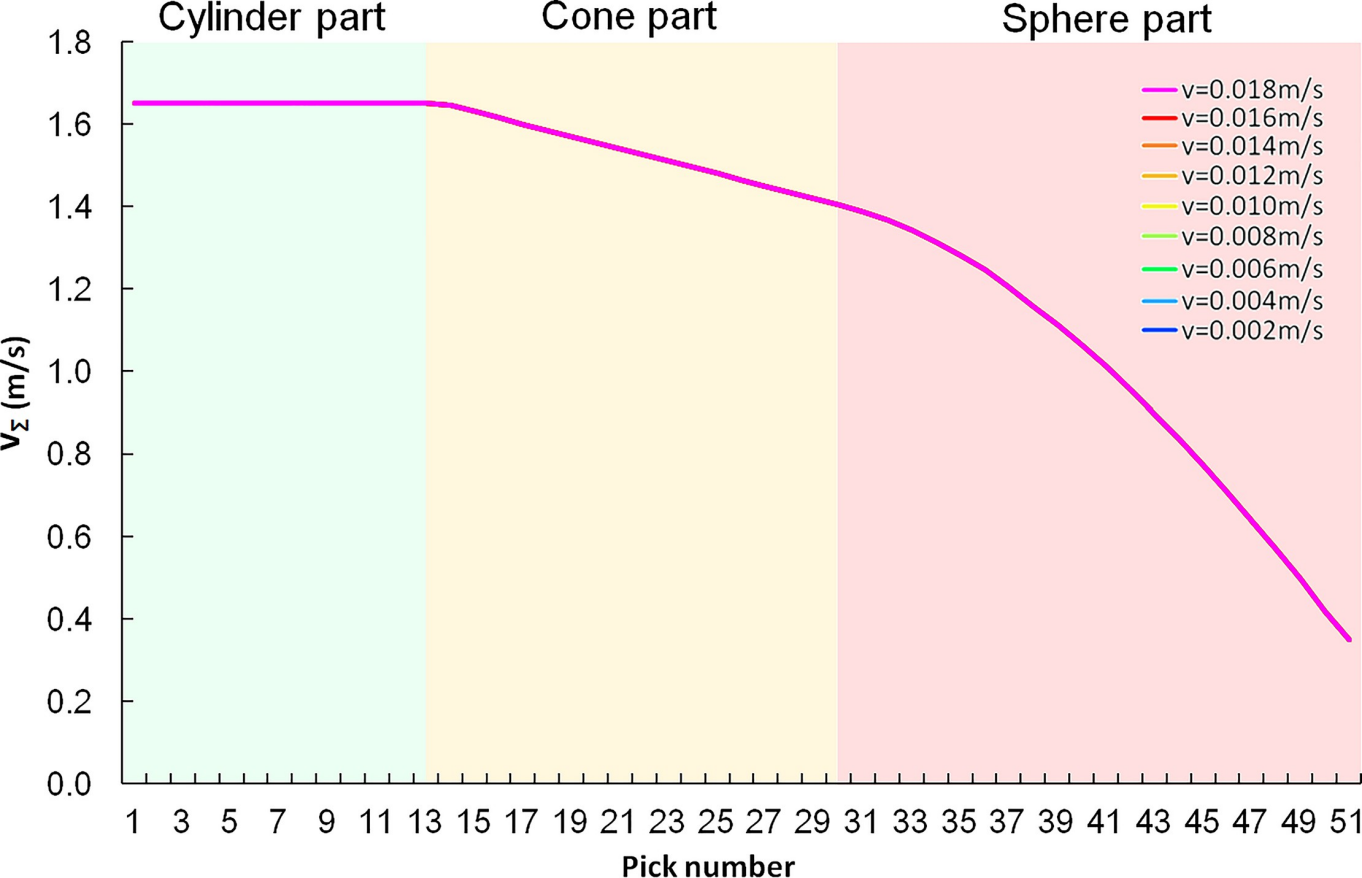
Cutting speed of each pick.

In rock cutting simulation, the swinging linear speed of each pick with the cutting head does not exceed 5.1% of the rotating speed; therefore, the rotating speed of the cutting head is the main factor causing the wear of the picks. The cutting speed of each pick is shown in Fig 20, and the cutting speeds at different swing speeds are close.

This paper proposes the pick failure index (PFI) to analyze the pick failure risk at different positions on the cutting head. The calculation method of PFI is in Eq(18).


$$PFI_{i}=\frac{CAI^{m_{F}}\times {\left(UCS/100\right)}^{n_{F}}\times \sqrt{({\left(K_{r}^{Avg}\right)}_{i}-K_{E})\times PCV_{i}\times 1000v_{\Sigma i}}}{S_{\mathrm{Ordinate}}}$$ (18)

In the equation, *S*_*Ordinate*_ is the ordinate scale coefficient and set to 10000 here. *CAI* value is set to 1 according to moderately abrasive. *UCS* is the value of uniaxial compressive strength, MPa. *m*_*F*_ and *n*_*F*_ is the influence coefficient of CAI and UCS, respectively, which are all set to 1. *K*_*r*_^*avg*^ is the average rotation coefficient in a circle. *K*_*E*_ is the expected value of the rotation coefficient, set to 1.22 for the longitudinal cutting head with three helices. The rest of the symbols are the same as before. Due to pick consumption is positively correlated with the cutting volume and the cutting speed; both are the main factors for the pick failure. As a result, the geometric mean of these two factors is used in the equation. Various cutting volumes or cutting speeds foreach pick on the cutting head will cause differences in pick failure. Due to the K_r_^avg^ being affected by the cutting groove’s geometry, K_r_^avg^ is used as the correction coefficient for PCV in the equation. CAI^n^ and (UCS/100)^n^ are used as the direct influence factors of PFI. As the CAI or UCS changes, the PFI value will change accordingly. However, the shape of the curves will not change; when m_F_ and n_F_ change, both the PFI value and curve shape will change.

**Fig 21:**
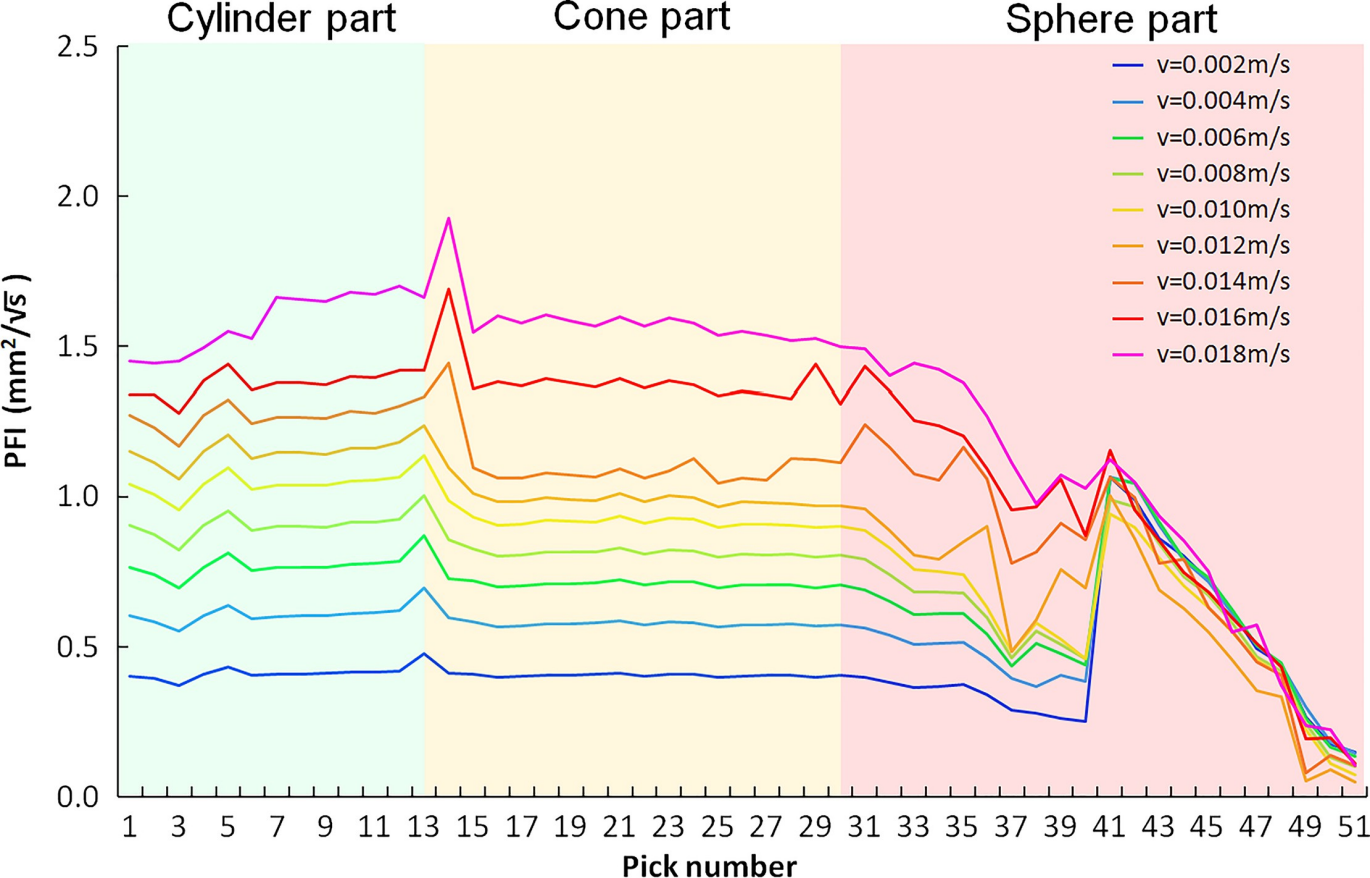
Pick failure model for the longitudinal cutting head.

The pick failure model for each pick on the cutting head with different speeds is shown in Fig 21. The pick failure risk on the cylinder is higher by the influence of cutting speed. In the transition region between the cylinder and cone, the cutting speed is still high. Due to the symmetry reduction of the cutting chip unit, the pick failure risk increases by the influence of the pick rotation coefficient. With reducing the pick rotation radius and cutting speed, the failure risk of picks on the cone gradually decreases. In the sphere of the cutting head, the PFI of picks in the area adjacent to the cone decreases with the decrease in cutting speed and the cutting volume. The PFI of picks on top of the sphere is sharply rise affected by the increase of rotation coefficient. When the swing speed is low, the pick failure risk of P41-P48 is significantly higher than other picks at this speed. As the swing speed increases, the PFI difference for all picks on the cutting head decreases. When the cutting head swings at high speed, the cutting groove depth increases, and the asymmetry influence of the P41 cutting groove decreases. The pick failure risk is higher with higher PFI due to the more considerable amount of cutting volume. Therefore, excessive swing speed should not be used in the cutting process to reduce pick consumption. For rocks with various UCS, CAI is different; however, the pick failure risk at each position on the cutting head is similar to the above curve.

In addition, it should be pointed out that this model is only applicable for pick failure prediction in a normal rock cutting state; and does not include excessive wear of picks on the start part of the cylinder. The excessive wear is usually caused by the cutting head continuously rotating in the loose cutting clips and cannot effectively dissipate heat while cleaning the roadway floor.

#### 3.4.2 Specific energy simulation

Rock cutting specific energy is the primary indicator to measure the cutting efficiency for roadheader. When cutting the rock with high UCS, the swing speed of the cutting head is slow due to the shallow depth of the cutting groove. Although the cutting power consumption is low, the corresponding specific energy is high, and the cutting head is in a low-efficiency cutting state. On the contrary, the specific energy will be reduced for rock with low UCS. The specific energy of rock cutting can be calculated by Eq (19),

$$SE=\frac{\int_{0}^{t}P(t)dt}{V_{C}}$$ (19)

where *SE* is specific energy, kWh/m^3^.*V*_*C*_ is rock cutting volume by the cutting head, m^3^.*t* is the cutting time, hour.

**Fig 22:**
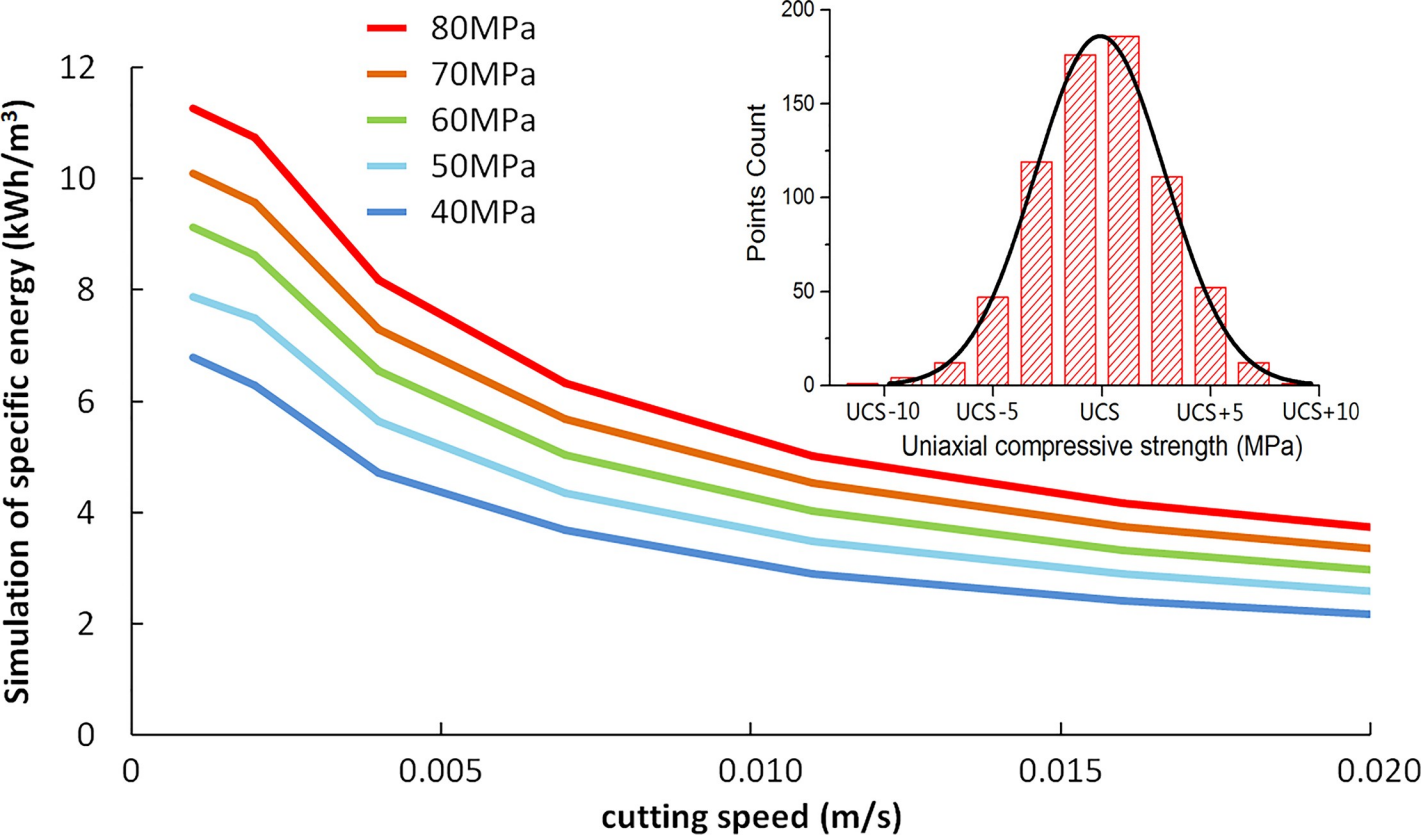
Calculation results of specific energy.

The specific energy is calculated within the design working capacity of EBZ260W roadheader. The specific energy is calculated with CH51 cutting heads completely drilled into the rock, and the cutting thickness is equal to the cylinder radius. The UCS for rocks is divided into five grades from 40MPa to 80MPa; the normal distribution random data is used in each pick calculation step to make the force on a single pick close to the actual cutting conditions. Furthermore, the calculation result of specific energy is close to the actual situation, as shown in Fig 22. The calculation results show that the specific energy decreases with the increase of cutting speed, and the specific energy of rock with high UCS is more significant.

## 4.Rock cutting verification

### 4.1 Artificial rock wall cutting test

**Fig 23:**
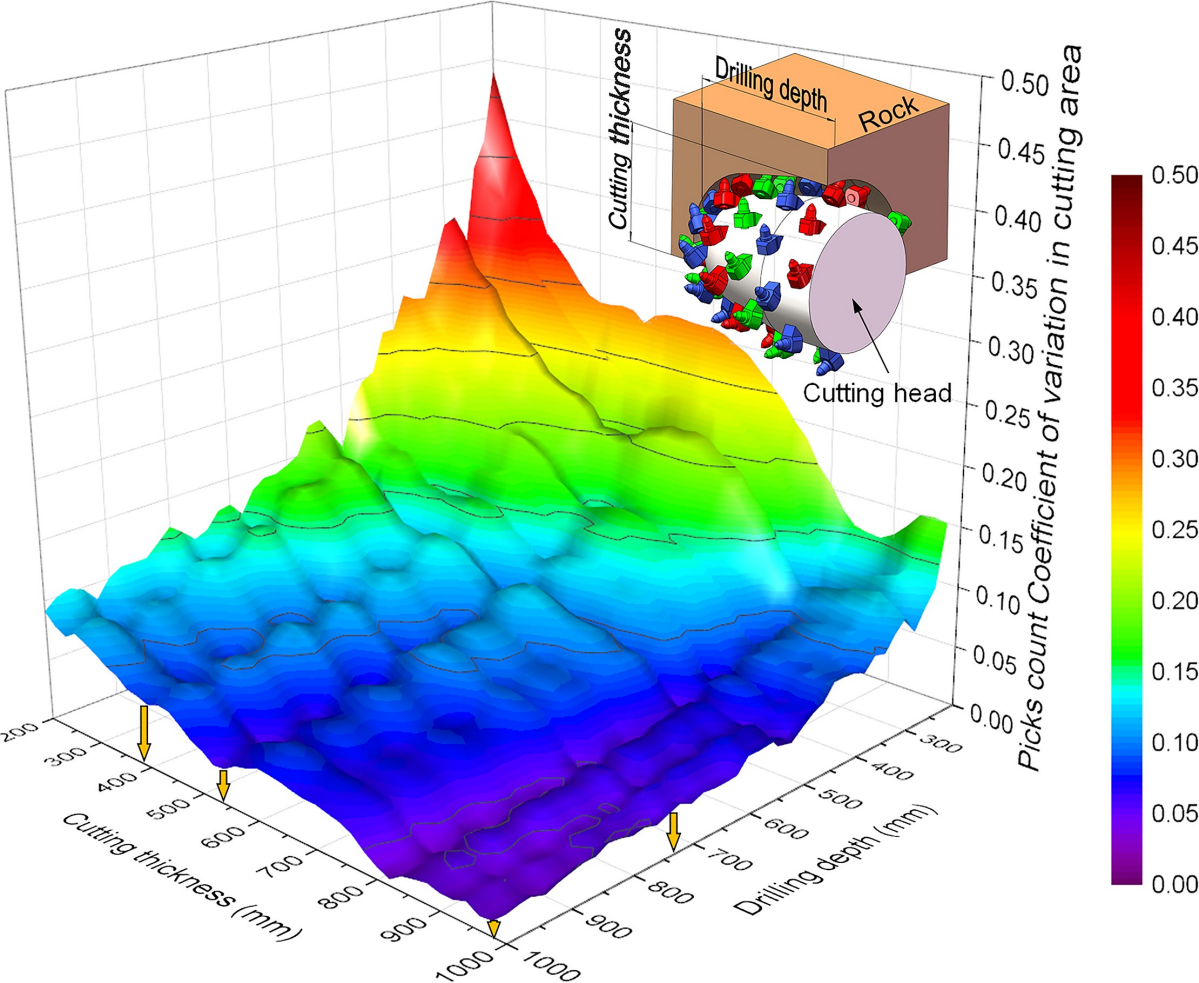
Cutting test parameters determined via drilling depth and cutting thickness.

In National Engineering Laboratory for Coal Mining Machinery, a group of cutting tests was performed on the CH51 cutting head with pick tip of φ25 tungsten carbide via EBZ260W roadheader. The artificial rock wall is composed of natural rock of sandstone and high-strength concrete; the UCS divided into three grades is 60MPa to 100MPa. The rock cutting test was carried out in the grade of 80MPa, and the UCS of the rocks has been measured to be 74±8MPa.To reduce the pick consumption caused by cutting load variation, the drilling depth and cutting thickness are analyzed to minimize the variation of pick count in the cutting zone, and the CV result of pick count is shown in Fig 23. With the increase of cutting depth and cutting thickness, the picks count in the cutting zone increases, and the corresponding CV decreases. When the cutting thickness is 380mm, 540mm, 820mm, 910mm and the whole cutting head is in the cutting zone, the CV of picks count in the cutting zone is smaller; and when the drilling depth is 500 mm, 680 mm, 740 mm and 930 mm, the CV of picks count is also smaller. Therefore, according to the artificial rock wall cutting in the laboratory, the drilling depth of the cutting head can be determined by 740mm, with the cutting thickness at 380mm, 540mm, and 970mm, respectively.

**Fig 24:**
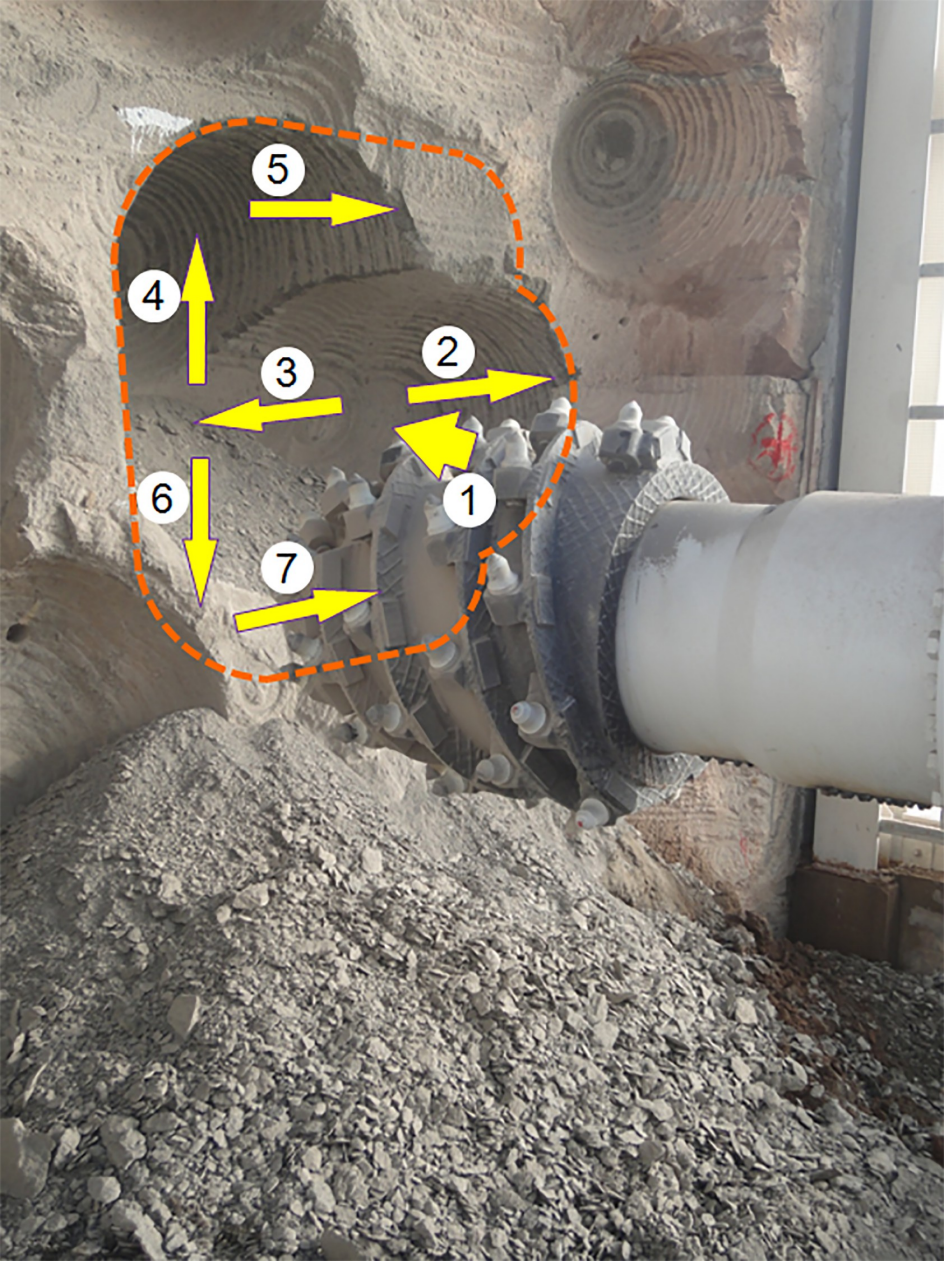
Artificial rock wall cutting test with EBZ260W roadheader.

The test process consists of 7 steps, as shown in Fig 24. After drilling into the artificial rock wall of 740mm (step 1), the cutting head moved to the right side (step 2)with the maximum cutting thickness of 970mm. At the same cutting thickness, the cutting head moved to the left side, then moved upward (step 3 and step 4), and then moved to the right side with 540mm cutting thickness (step 5). Finally, after moving downward with the cutting thickness of 970 (step 6), the cutting head moved to the right side with the cutting depth of 380mm (step 7).

### 4.2 Statistics of pick consumption

**Fig 25:**
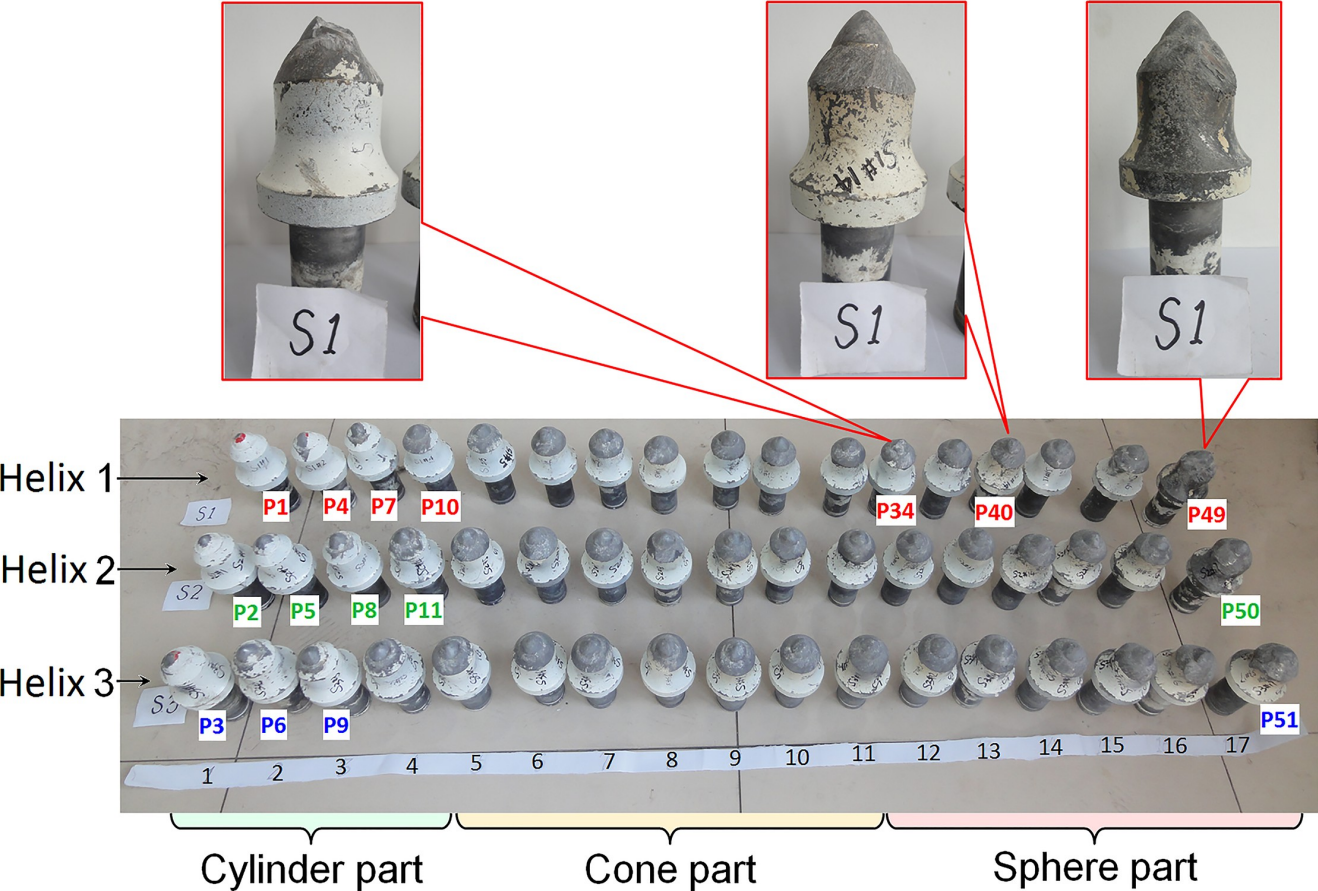
Picks of rock cutting test.

The pick wear of 22mm tungsten carbide tip is shown in Fig 25. Among all picks on the cutting head, picks of P11 to P51 were in the cutting zone. The mass loss and height loss of P34 at position 12 on helix 1 are more significant due to the breakage of the tungsten carbide. On the other hand, more metal wear can be observed on the conical shank of P40 and P49 at positions 14 and 17 on helix 1. However, the tungsten carbide of the pick tip was not blunt, and the picks were still in regular use.

The height loss of the pick is mainly used to describe the wear of the top tungsten carbide; the mass loss is used to describe the loss of the tungsten carbide and the wear of the entire pick. Both the height loss and the mass loss have a significant impact on the working life of the picks. This article proposes to use the equivalent loss to analyze the actual loss of the pick. The equivalent loss is calculated by Eq (20),

$$M_{HL}=\sqrt{M_{L}•H_{L}}$$ (20)

where *H*_*L*_ is the height loss, mm.*M*_*L*_ is the mass loss, g. *M*_*HL*_ is the equivalent mass loss,(mm·g)^1/2^.

**Fig 26:**
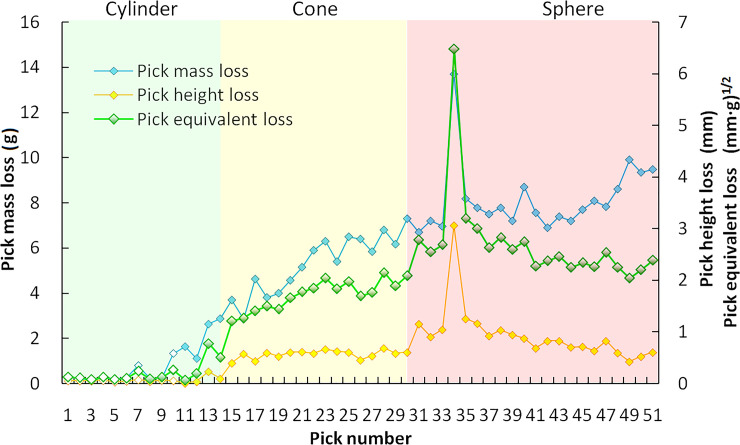
Pick consumption of CH51 cutting head.

The cutting volume calculation results show that from bottom to top of CH51 cutting head, as the cutting chip unit area decreases, the volume of directed rock cutting by a pick in the cutting zone gradually decreases. Especially in the sphere with a large number of picks, the rock cutting volume decreases linearly; however, the theoretical cutting volume of P34 did not change significantly. Therefore, the abnormality of equivalent loss caused by thetungsten carbide broken of P34 is related to the extremely high hardness of the local rock mass or the manufacturing defect of the tungsten carbide itself, which is a random interference factor. For all picks in the cutting zone, the mass loss increases with the axial position, and the height loss gradually decreases on the sphere, as shown in Fig 26. In the swing process, the cutting depth of each pick increases with the swing radius increase, and the mass loss caused by the conical shank wear of the pick increases correspondingly. However, the height loss is not significant, and the equivalent loss is increasing due to the influence of mass loss. On the sphere, the mass loss caused by the metal wear of the conical shank continues to increase with the symmetry decrease of the cutting clip unit caused by the increase of the skew angle. However, due to the higher density of pick arrangement on top of the sphere and the small cutting line spacing, each pick can effectively share the cutting force on the adjacent picks, reducing the consumption of the tungsten carbide, thereby reducing the equivalent loss.

In the test, only one pick on the cylinder cut the rock with the free surface. Therefore, the correlation between PFI and the actual pick failure on cone and sphere is analyzed. During the whole test process, the average swing speed is low at 0.003m/s. The cutting depth of each pick on the cone is from 4.5 to 5.5mm, and the actual cutting volume is similar. The height loss changes slightly, and the mass loss increases with the symmetry decrease of the cutting groove caused by the increase of the cutting depth. The PFI calculation results are consistent with the rock cutting test results, and the correlation coefficients of high loss and mass loss are 0.70 and -0.74, respectively. For the picks on the sphere adjacent to the cone, the correlation coefficients between PFI and pick height loss and mass loss are 0.74 and -0.81, respectively. For the picks on top of the sphere, the PFI is significantly affected by the cutting speed. However, both PFI and the actual height loss show a decreasing trend for the reduction of cutting volume. The correlation coefficients of high loss and mass loss are 0.77 and -0.91, respectively.As a result, the pick consumption model can be used for the longitudianl cutting head.

**Fig 27:**
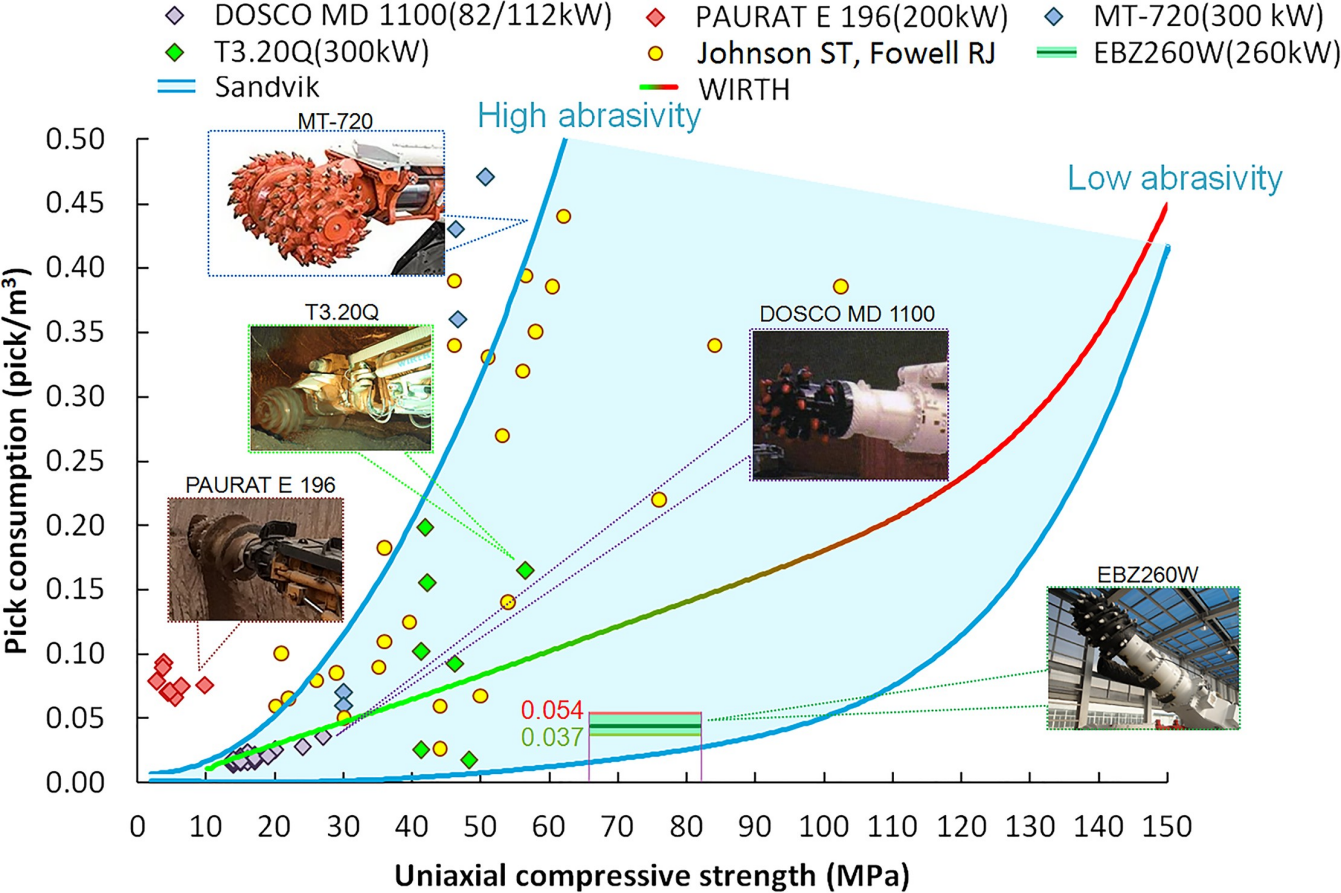
Comparison of pick consumption.

Taking the pick box is non-contact with the rock as the constraint, the theoretical maximum value of the pick height loss can be up to 11mm, and the corresponding mass loss is 70g. The mass loss and height loss per unit volume of picks are 0.054 and 0.037, and the sum of cutting volume is 1.77m^3^. The average pick consumption calculated by the equivalent loss is 0.044 picks/m^3^, which is lower than the pick consumption of the general cutting heads [30–35], as shown in Fig 27. For the rock with UCS from 66MPa to 82MPa, the pick consumption of the CH51 cutting head based on the pick arrangement meshing method meets statistical results on pick consumption of roadheader from Sandvik engineering verification with φ22 tungsten carbideand is at a low consumption level [36]. Compared with the pick consumption statistical data of WIRTH typical cutting head [37],the pick consumption of CH51 cutting head is only 37% of the statistical results with φ22 tungsten carbide. CH51 cutting head of EBZ260W roadhead used improved tungsten carbidewithφ25 diameter, improved pick arrangement on three helices, and the small diameter of cutting head for rock cutting are the main reason for the lower pick consumption. In addition, the optimized cutting depth and cutting thickness are other reasons to reduce the fluctuation of cutting load and reduce the pick consumption.However, in the actual roadway cutting process, to avoid the wear of the pick box caused by pick height loss, the pick that does not reach the wear limit will be replaced in advance, which will increase the actual pick consumption. As a result, the on-site pick consumption will be higher than that of the artificial rock cutting test.The on-site pick consumption in roadway excavation will further modify the above test results.

### 4.3 Geometry of cutting clips

**Fig 28:**
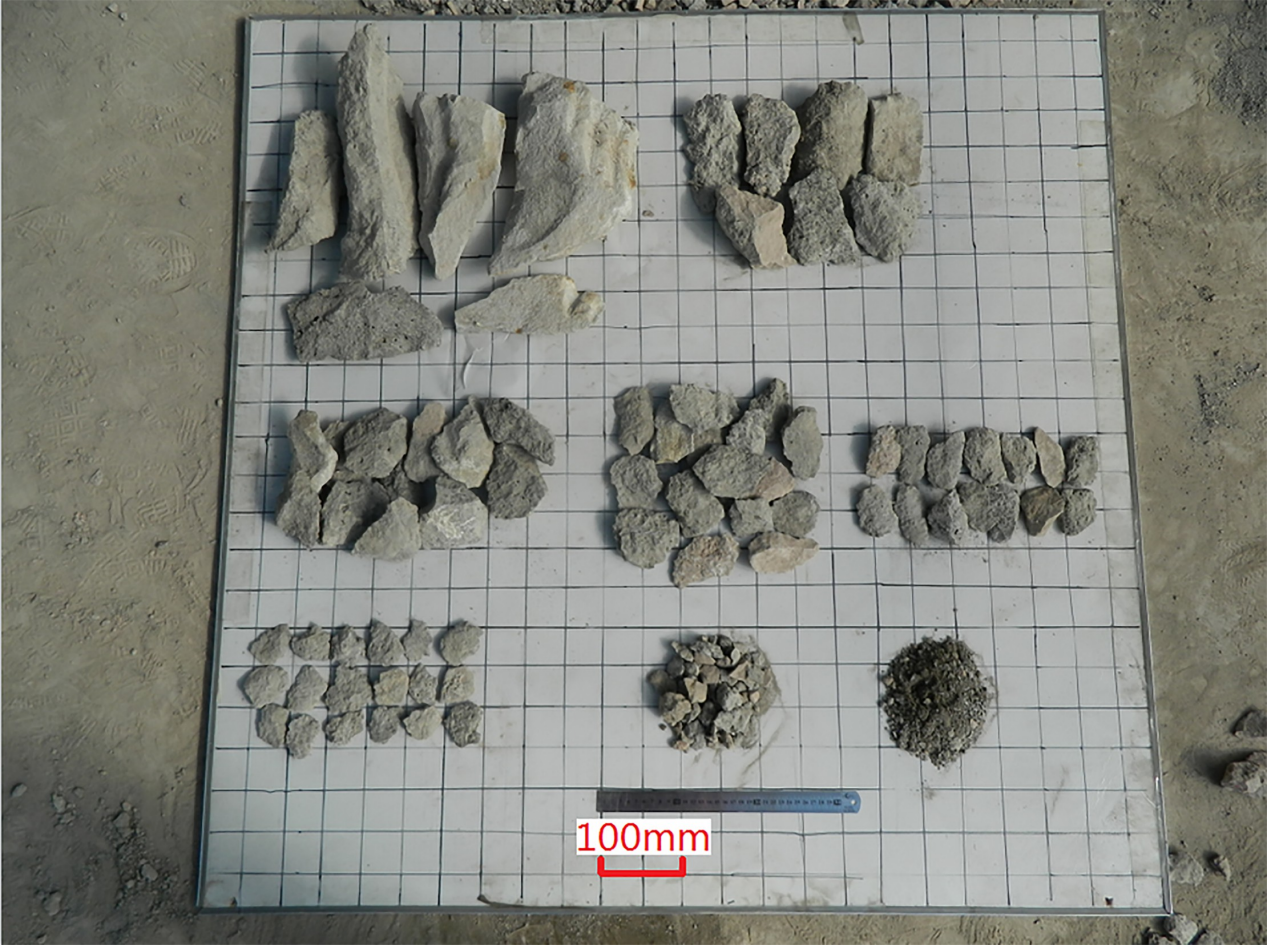
Typical geometry of chips.

The typical chip shape obtained in the test is shown in Fig 28. The chip length is determined by the cutting head rotating speed, swing speed and helix spacing. The helix spacing is the main factor affecting the chip width, and the cumulative cutting depth determines the chip thickness. When the cutting head moves in the vertical direction, the roadheader can be stably supported on the ground, and the swing speed is determined by the moving speed of the lifting cylinder. When the swing speed matches the accumulated cutting groove formed by the adjacent picks on the same helix, the chips will be a long strip with a larger size under the co-cutting action of the picks on the adjacent helix. Due to the friction torque between the roadheader and the ground is not enough to balance the rotating torque generated by the rotary cylinder in horizontal moves, the roadheader slips laterally, resulting in the accumulative cutting depth was shallow and the chip reduced.

**Fig 29:**
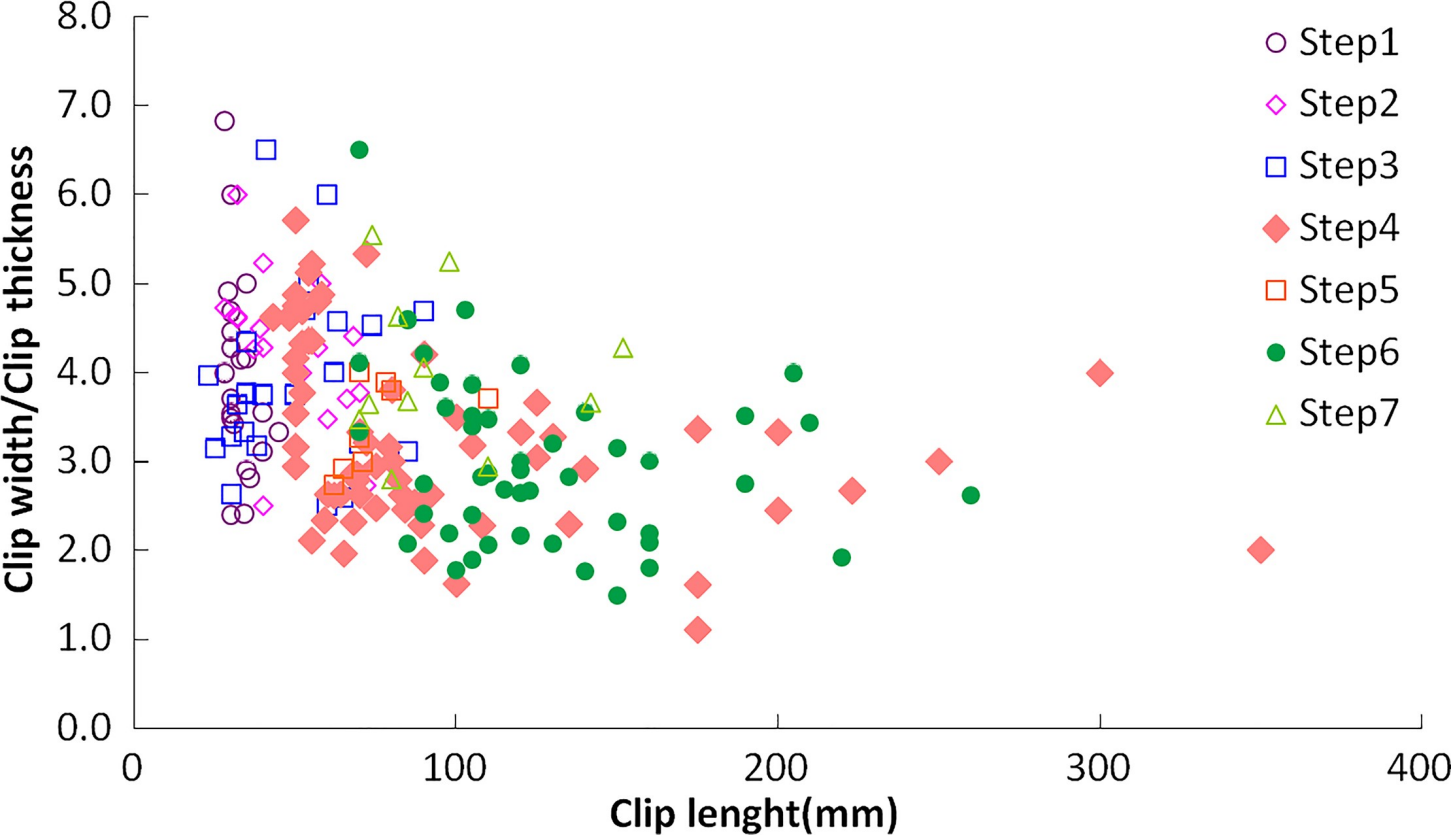
The ratio of chip width to chip thickness.

The statistical results of the chip geometry of Step1 to Step7 are shown in Fig 29. With the increase of the swing speed of the cutting head, the chip size increases. The chip length of Step4 and Step6 is more significant, and the chip length of drilling of Step1 is generally smaller. Since there is no free surface in Step2 and Step3, the chip length is relatively smaller; while in the cutting process of Step5 and Step7, the chip length is more significant due to the free surface excavated in Step2 and Step3. With the influence of rock mass bedding and joint direction, the chip width to chip thickness ratio is mainly concentrated in the range of 1.5–5.5.Among the randomly selected samples with the minimum length of chips exceeding 25mm, the amount of chips in the region of optimum specific energy is 86.1% to the total samples.

### 4.4 Specific energy of rock cutting

The results of each test step are shown in Table 5. In step 4 bottom-up cutting process, the roadheader obtains stable support without the influence of rotating torque, the cutting speed is the highest, and the cutting power is close to the rated power. Therefore, the cutting efficiency is highest. In addition, due to the equipment connection failure, the cutting power of Step1 and Step3 was not available.

**Table 5:** Results of each cutting step.

Results	Artificial rock wall cutting step						
	Step1	Step2	Step3	Step4	Step5	Step6	Step7
Mean swing speed(m/s)	4.2×10^−3^	9.8×10^−4^	1.4×10^−3^	6.3×10^−3^	1.5×10^−3^	2.3×10^−3^	2.4×10^−3^
Cutting volume(m^3^)	0.373	0.259	0.315	0.256	0.213	0.188	0.165
Peak power(kW)	−	70.7	−	254.1	116.5	117.3	86.3
Mean power(kW)	−	49.9	−	128.1	50.8	88.8	40.3

**Fig 30:**
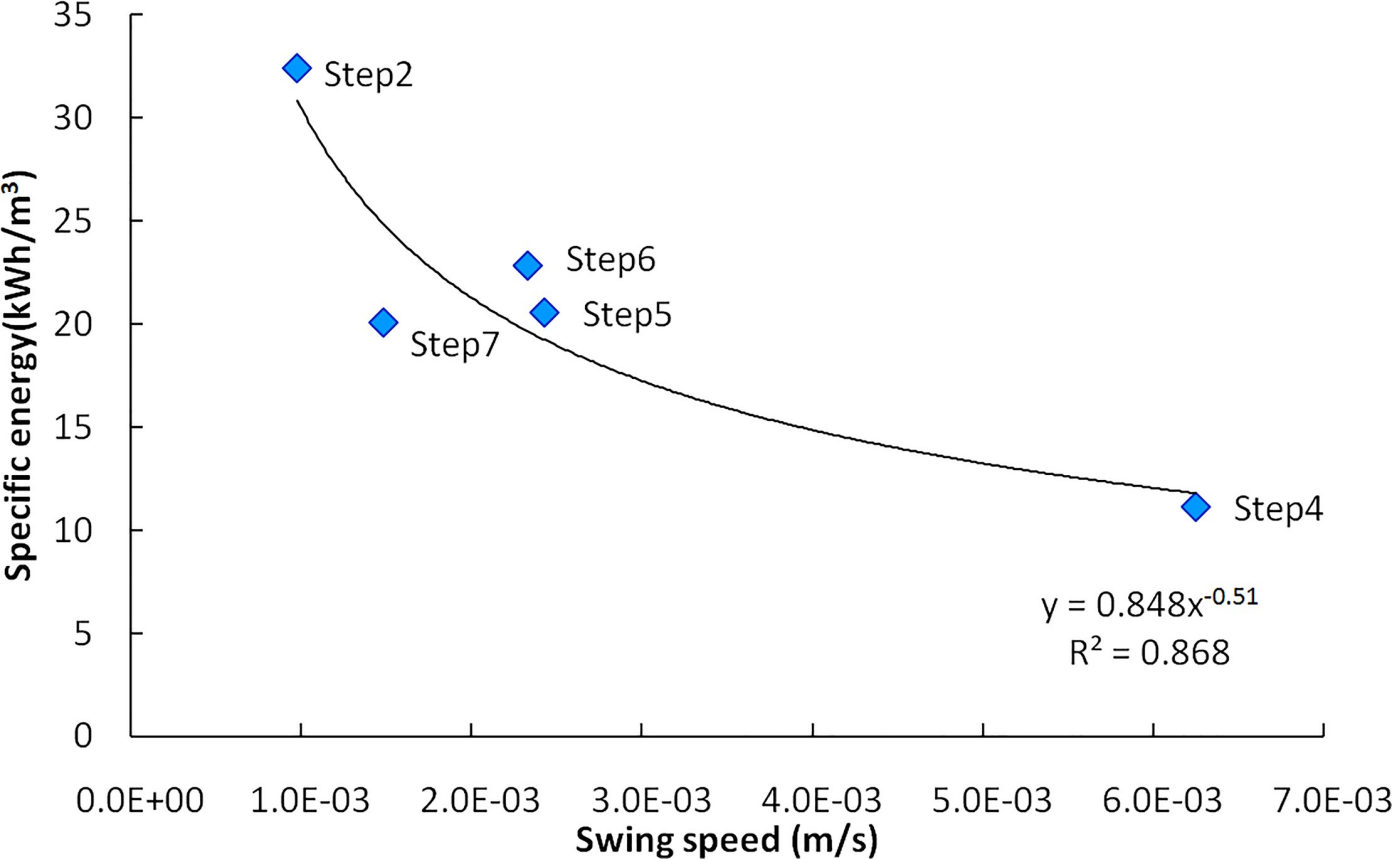
Rock cutting specific energy.

**Fig 31:**
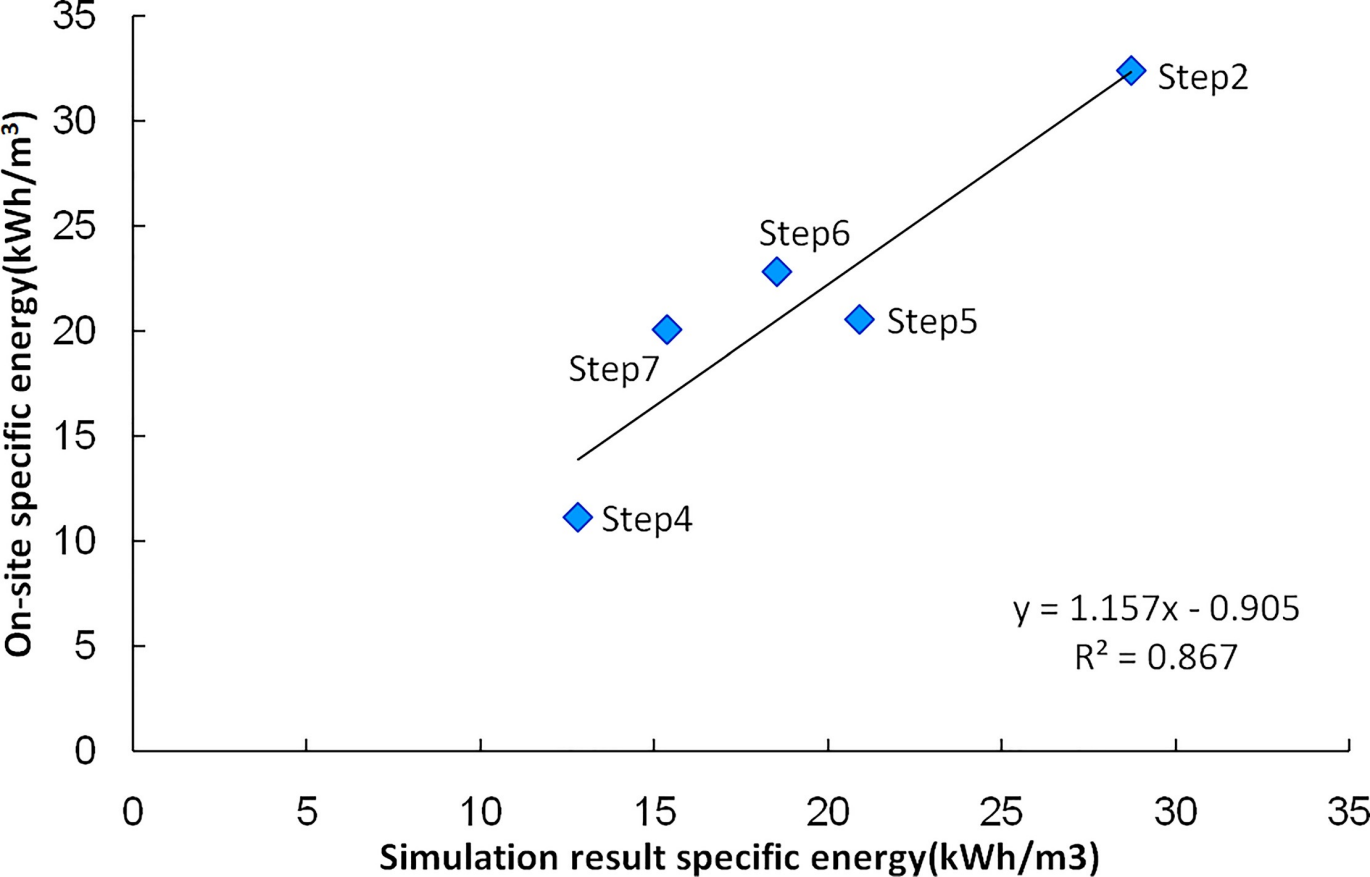
Specific energy relationship between on-site test and simulation result.

The cutting power increases with the swing speed. The rock cutting specific energy for each test step is shown in Fig 30. As the swing speed increases, the specific energy decreases gradually. The cutting specific energy of the step4 with the highest cutting speed is only 1/3 of Step2; by the free surface of Step2 and Step3, the specific energy of Step5 and Step7 are significantly lower with the same cutting conditions. The simulation results of each cutting step using the rock cutting test parameter show that the on-site specific energy is linearly related to the simulation results. The simulation results can appropriately reflect the working performance of the cutting head, and the correlation coefficients between on-site specific energy and calculate resultis 0.93, as shown in Fig 31.

In addition, the rock cutting test results show that cutting rocks with a swing from bottom to top can effectively reduce the specific energy and improve the cutting effect for hard rocks. The swing path with free surfaces should be employed for sequential cutting in the on-site rock cutting process.

## 5 Conclusions

The pick arrangement meshing method based on the traversal algorithm is suitable for the pick arrangement of cutting heads that cannot be directly expanded into a plane. The rock cutting test results show that the cutting head optimized by this method can reduce pick consumption and improve the working performance. The conclusions of this study are as follows:

1.The method based on meshing the spatial position of the picks can optimize the pick arrangement parameters of the cutting head. The TMR indicators composed of mesh skewness, mesh symmetry, and mesh area ratio can be used to analyze the uniformity of pick arrangement. For the longitudinal cutting head with cylinder, cone, and sphere, the TMR and pick arrangement uniformity of 3-helix are higher than those of 2-helix and 4-helix cutting heads. The ratio of helix spacing to the cumulative cutting depth of cutting head with 3-helix is concentrated from 2 to 5 and consistent with the optimum specific energy. In the range of normal cutting depth, the rotation coefficient of picks is between 1.05–1.40 with slight variation, and suitable for various rotation speed and swing speed, which indicate that the optimization method based on pick position meshing method is reasonable.

2.The standard mesh of the optimized CH51 cutting head is mainly concentrated on the cylinder and cone. The average value of pick tip distance to the average value of the helix spacing is 1.4, and the TMR reaches 53.19%. As a result, the pick arrangement is uniform, and the variation of cutting load with different cutting thicknesses is lower. Based on cutting speed, direct rock cutting volume of each pick, pick rotation efficiency, uniaxial compressive strength, and CERCHAR abrasivity index, the pick failure model of the cutting head shows that under normal rock cutting conditions, for low swing speed, the failure risk of picks on top of the cutting head is higher than that of other picks; as the swing speed increases, the pick failure risk of the cylinder and cone increases. The specific energy decreases with the increase of swing speed.

3. The cutting test results on artificial rock walls show that the mass loss of the longitudinal-axis cutting head increases with the cutting depth; the pick height loss decreases with the reduction of the direct rock cutting volume by the picks. Since the height loss is accompanied by mass loss, the equivalent loss is more significantly affected by the height loss. PFI is positively correlated with the height loss of the pick and negatively correlated with the mass loss. The correlation gradually increases from the cone to the sphere. The pick consumption in the rock cutting test is 0.037–0.054 picks per cubic meter, which shows that the pick arrangement results based on meshing the spatial position of the picks are reasonable, and the rock cutting test parameters are selected appropriately. The simulation results of specific energy have a linear relationship with the actual results, which can be used to analyze the working performance of the cutting head at different swing speeds.

## Supporting information

S1 Data Mesh cell of CH51cutting head. (XLSX)

S2 Data TMR of CH51 cutting head. (XLSX)

S3 Data Pick consumption of CH51 cutting head. (XLSX)

S4 Data Specific energy. (XLSX)
